# RAB6 and microtubules restrict protein secretion to focal adhesions

**DOI:** 10.1083/jcb.201805002

**Published:** 2019-05-29

**Authors:** Lou Fourriere, Amal Kasri, Nelly Gareil, Sabine Bardin, Hugo Bousquet, David Pereira, Franck Perez, Bruno Goud, Gaelle Boncompain, Stéphanie Miserey-Lenkei

**Affiliations:** 1 Dynamics of Intracellular Organization Laboratory, Institut Curie, PSL Research University, Sorbonne Université, Centre National de la Recherche Scientifique, UMR 144, Paris, France; 2 Molecular Mechanisms of Intracellular Transport Laboratory, Institut Curie, PSL Research University, Sorbonne Université, Centre National de la Recherche Scientifique, UMR 144, Paris, France

## Abstract

To ensure their homeostasis and sustain differentiated functions, cells continuously transport diverse cargos to various cell compartments and in particular to the cell surface. Secreted proteins are transported along intracellular routes from the endoplasmic reticulum through the Golgi complex before reaching the plasma membrane along microtubule tracks. Using a synchronized secretion assay, we report here that exocytosis does not occur randomly at the cell surface but on localized hotspots juxtaposed to focal adhesions. Although microtubules are involved, the RAB6-dependent machinery plays an essential role. We observed that, irrespective of the transported cargos, most post-Golgi carriers are positive for RAB6 and that its inactivation leads to a broad reduction of protein secretion. RAB6 may thus be a general regulator of post-Golgi secretion.

## Abstract (precis)

Fourriere et al. demonstrate the existence of secretion hotspots juxtaposed to focal adhesions. Post-Golgi transport carriers use a subset of microtubules to reach focal adhesions. RAB6 acts as a general regulator of post-Golgi secretion and, together with ELKS, restricts protein secretion at focal adhesions.

## Introduction

To reach the cell surface, secreted proteins are transported along intracellular routes from the endoplasmic reticulum (ER) through the Golgi complex. Cargos exit the Golgi complex in transport carriers that use microtubules to be addressed rapidly to the plasma membrane before exocytosis. Transmembrane proteins are then exposed at the plasma membrane while soluble cargos are released in the extracellular space. Whether delivery of cargos occurs randomly or at specific sites of the plasma membrane is still unclear, and the mechanisms that direct exocytosis are still unknown. Microtubules were described to be captured and stabilized by focal adhesions (Kumar et al., 2009). Their targeting to focal adhesions is driven by microtubule plus-end tracking proteins, such as adenomatous polyposis coli, end-binding protein, and cytoplasmic linker-associated protein (CLASP), which ensure their physical contacts (Lansbergen et al., 2006; Akhmanova and Steinmetz, 2008; Kumar et al., 2012; Stehbens et al., 2014). Additionally, microtubules are linked to the actin network, which is a structural component of focal adhesions (FAs; Palazzo and Gundersen, 2002). Notably, microtubules are involved in the regulation of the distribution and dynamics of adhesion sites (Small et al., 2002; Stehbens and Wittmann, 2012; Etienne-Manneville, 2013).

CLASPs interact at the plasma membrane with a protein complex made of LL5β, a phosphatidylinositol 3-phosphate–binding protein, and ELKS (also named ERC1 for ELKS/Rab6-interacting/CAST family member 1; also known as RAB6IP2). ELKS is an effector of the Golgi-associated Ras-related protein 6 (RAB6) GTPase (Monier et al., 2002), which regulates several anterograde and retrograde trafficking pathways to and from the Golgi complex, as well as Golgi homeostasis (Goud, 1999; White et al., 1999; Mallard et al., 2002; Grigoriev et al., 2007). In particular, RAB6 was shown to be involved in the targeting of post-Golgi vesicles containing the secretory markers vesicular stomatitis virus glycoprotein (VSV-G; a type I transmembrane protein), and neuropeptide Y (NPY; a soluble protein) to ELKS-enriched regions of the plasma membrane (Miserey-Lenkei et al., 2010; Grigoriev et al., 2011). RAB6 has been also shown to regulate the secretion of TNFα in macrophages (Micaroni et al., 2013) and the trafficking of herpes simplex virus 1 (Johns et al., 2014). Herpes virus particles were shown to be associated with the RAB6 machinery in infected cells, and their exocytosis was observed to occur in close proximity to LL5β patches (Hogue et al., 2014). However, thus far, no systematic study has been performed to characterize the cargos present in RAB6-positive vesicles.

The aim of this study was to investigate the spatial organization of post-Golgi trafficking of a variety of anterograde cargos in nonpolarized cells. To this end, we combined the retention using selective hooks (RUSH) assay (Boncompain et al., 2012) to synchronize anterograde transport of cargos and a selective protein immobilization (SPI) assay to map precisely the sites of arrival of the cargos at the plasma membrane. We show that cargos are transported along microtubules to hotspots of secretion, which are juxtaposed to FAs. Moreover, we found that RAB6-dependent post-Golgi machinery plays a key role in this process and that RAB6 could be a general regulator of post-Golgi secretion.

## Results

### Exocytosis takes place in restricted areas, close to the adhesion sites

**Figure 1.**
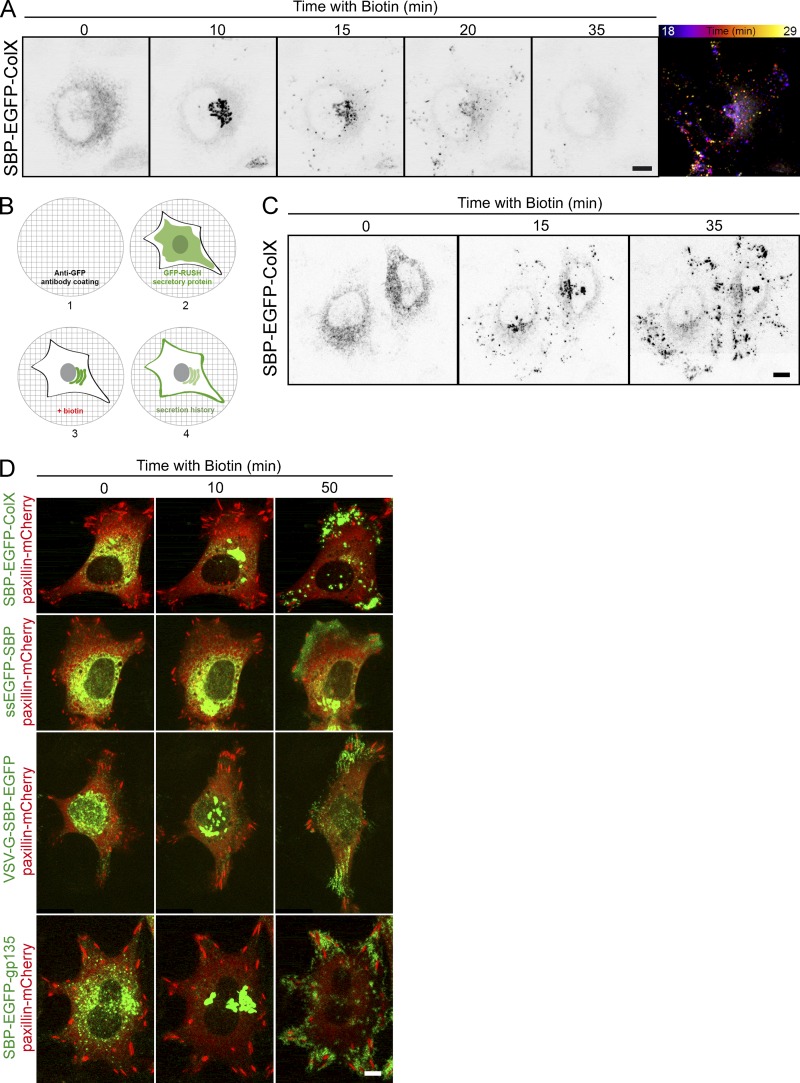
**Local exocytosis close to adhesion sites of the cells. (A)** HeLa cells stably expressing SBP-EGFP-ColX were incubated with biotin for the indicated time (min). Real-time pictures were acquired using a spinning disk microscope at the indicated times. Temporal projection (right image) was performed for the SBP-EGFP-ColX signal between 18 and 29 min of trafficking. **(B)** Description of the SPI assay. (1) A coverslip is coated with an anti-GFP antibody. (2) The GFP-RUSH cell line is seeded on the coverslip. (3) Addition of biotin allows the trafficking of the GFP-RUSH cargo. (4) Interactions between the anti-GFP and the GFP of the cargo (transmembrane or secreted) allow the capture of the cargo and provide a picture of the history of the secretion. **(C)** Trafficking of SBP-EGFP-ColX with an anti-GFP coating (SPI assay). HeLa cells stably expressing SBP-EGFP-ColX were incubated with biotin for the indicated time. Real-time images were acquired using a spinning disk microscope at the indicated times. **(D)** HeLa cells were transfected with SBP-EGFP-ColX, ssEGFP-SBP, VSV-G-SBP-EGFP, or SBP-EGFP-gp135, and paxillin-mCherry. Coverslips were coated with an anti-GFP coating (SPI assay). Cells were observed by time-lapse imaging using a spinning disk microscope, and pictures were acquired at the indicated times. Scale bars, 10 µm.

Secretion of newly synthesized proteins along the secretory pathway occurs continuously in cells. The RUSH system offers the possibility to synchronize the intracellular transport of cargos fused to the streptavidin-binding peptide (SBP) upon addition of biotin in the culture medium (Boncompain et al., 2012). With this system, it is possible to monitor a wave of secretion of a selected cargo and analyze its transport to the cell surface. Using the RUSH assay, we studied the synchronous secretion of diverse cargos: collagen type X (ColX), VSV-G, secretory soluble EGFP (ssEGFP), gp135 (podocalyxin), the glycosylphosphatidylinositol-anchored proteins (GPI-APs) cluster of differentiation 59 (CD59) and placenta alkaline phosphatase (PLAP), and TNFα. Fig. 1 A illustrates RUSH-based transport monitoring using ColX as a cargo. As expected, before biotin addition, ColX was retained in the ER (Fig. 1 A, 0 min). Upon biotin addition, ColX left the ER, reached the Golgi apparatus within 10 min postrelease, and was then exocytosed at the plasma membrane. About 35 min after biotin addition, most of ColX had been secreted into the medium, and almost no signal remained in cells. Time-lapse imaging and temporal projection after Golgi exit suggested that exocytosis did not occur randomly at the cell surface but in preferred domains (Fig. 1 A and Video 1). However, because ColX is a soluble secretory protein, a significant fraction of released proteins diffuses out, which may lead to underestimate levels of exocytosis at these preferred sites. To prevent its diffusion after release, we set up an assay that we named SPI. In this assay, a GFP moiety is fused to soluble secretory factors or to the luminal part of membrane-bound cargos, and before seeding the cells, coverslips are coated with anti-GFP antibodies (Fig. 1 B). The interaction between the coated anti-GFP antibodies and the GFP moiety fused to the cargos reduces the diffusion speed of the cargos and eventually immobilizes them. This enables the local accumulation of secreted proteins that were released over an extended period of time.

We showed that the coated antibodies are homogeneously distributed, enabling the SPI assay to quantitatively immobilize GFP-fused proteins (Fig. S1). In combination with the RUSH assay, a complete overview and localized history of the secretion of a selected cargo is obtained. As shown in Fig. 1 C and Video 2, the use of the SPI enables a strong accumulation of secreted ColX (see the differences between Fig. 1 A, without SPI, and Fig. 1 C). The presence of hotspots of ColX secretion confirmed that some domains of the plasma membrane seemed unable to support exocytosis, while others were very active. The localization of the active domains was reminiscent of FA sites. We used cells expressing paxillin-mCherry, which localizes to FAs (Turner et al., 1990; Turner, 1998), to monitor the synchronized transport of ColX combined with SPI and found that secreted ColX was clearly enriched on FAs (Fig. 1 D). A similar result was obtained for another soluble cargo, ssEGFP, although it appeared more diffuse when secreted, likely due to more rapid diffusion than ColX and/or less efficient capture by the antibody (Fig. 1 D).

Similar experiments were performed with membrane-bound cargos such as VSV-G, gp135, TNFα, and E-cadherin adapted to the RUSH assay. Although no particular enrichment was observed in normal conditions (Fig. S1), probably due to a rapid diffusion of secreted cargos in the plane of the plasma membrane, topologically restricted secretion was observed using SPI. As for secreted cargos, exocytosis of VSV-G and gp135 also occurred on hotspots localized to FAs (Fig. 1 D). The same results were obtained when monitoring E-cadherin and TNFα secretion (data not shown). The combination of the RUSH and SPI assays thus demonstrated the existence of secretion hotspots close to FAs for soluble and membrane-bound proteins.

### Exocytosis is directed between FAs

**Figure 2.**
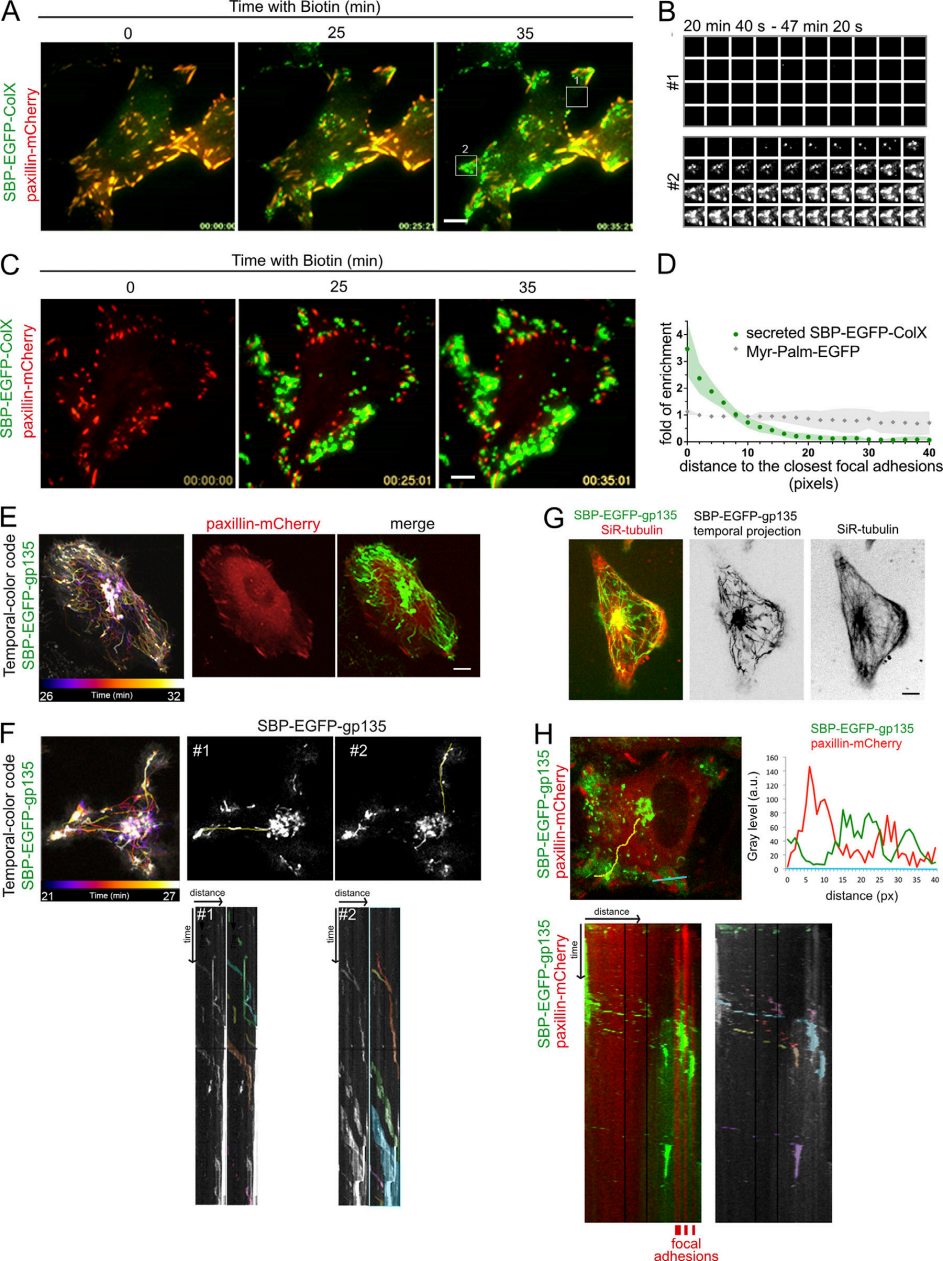
**Exocytosis is directed between FAs. (A)** HeLa cells were transfected with SBP-EGFP-ColX and paxillin-mCherry. Real-time pictures were acquired using a TIRF microscope at the indicated times. The TIRF angle was chosen based on the paxillin-mCherry signal. Biotin was added at time 0. **(B)** Fixed images of areas 1 and 2 (see A) taken every 40 s between 20 min 40 s and 47 min 20 s of ColX trafficking. Area 1 is a control condition without ColX signal at the plasma membrane. Area 2 shows secretion in close proximity to FAs. **(C)** Coverslips were coated with an anti-GFP antibody (SPI assay). Real-time images were acquired using a TIRF microscope at the indicated times. The TIRF angle was determined based on the paxillin-mCherry signal. Biotin was added at time 0. **(D)** Distance of secreted SBP-EGFP-ColX (green) or myristoylated and palmitoylated (MyrPalm)-EGFP (gray) signal from the closest FAs was measured. The distance of each pixel from the whole cell area from the closest FA was also measured. Ratio of enrichment compared with the distance of pixels of the region of interest to the closest FAs was calculated (*n* = 585 FAs, 10 cells for secreted SBP-EGFP-ColX and *n* = 319 FAs, 11 cells for Myr-Palm-EGFP). **(E–H)** HeLa cells were transfected with SBP-EGFP-gp135 (F and G) or SBP-EGFP-gp135 and paxillin-mCherry (E and H). Coverslips were coated with an anti-GFP antibody (SPI assay; H). Microtubules were stained with SiR-tubulin (G). Cells were observed by time-lapse imaging using a spinning disk microscope and images were acquired at the indicated times. Biotin was added at time 0. After 20 min of incubation with biotin, SBP-EGFP-gp135 localizes in the Golgi apparatus, and fast acquisition imaging was performed. Between 26 and 32 min (E) or 21 and 27 min (F), a temporal projection of EGFP-gp135 signal was performed using Fiji software and is represented with a temporal-color code (left). In F–H, kymographs (time space plots) were performed on the indicated yellow lines using Fiji software. In H, intensity profiles of SBP-EGFP-gp135 (in green) and paxillin-mCherry (in red) were performed using Fiji software at the indicated blue line. To help the interpretation of the kymographs, the passage of distinct post-Golgi carriers has been colorized. px, pixels. Scale bars, 10 µm.

Next, real-time analysis of exocytosis was performed using total internal reflection fluorescence (TIRF) microscopy. In agreement with results obtained with the SPI setup, we detected the frequent occurrence of exocytic events in certain regions of the plasma membrane, while other zones were seemingly silent (Fig. 2, A and B). Moreover, dual-color TIRF microscopy combined with SPI confirmed that exocytic events are enriched close to FAs and revealed that not all FAs are secretion hotspots (Fig. 2 C). We quantified the enrichment of secreted SBP-EGFP-ColX close to FAs by image analysis using the homogeneously distributed protein myristoylated and palmitoylated EGFP as a reference marker (Fig. 2 D).

To explain how such a restriction of exocytosis may occur, we envisioned two nonexclusive hypotheses. On one hand, directed transport to FAs may bias release toward adhesion domains. On the other hand, the factors essential to sustain exocytosis may be present only at the hotspots. To test the first hypothesis, we performed fast imaging (using a ∼200-ms frame rate) of synchronized post-Golgi transport of gp135 to detect potential privileged tracks that may direct transport toward hotspots. Temporal projections revealed that gp135 en route from the Golgi complex to the cell surface used direct tracks toward FAs (Fig. 2, E and F). Interestingly, when using poly-l-lysine as a substrate on which FAs do not form properly, no direct tracks to FAs were detected (Fig. S2). Microtubules are involved in the regulation of the distribution and dynamics of adhesion sites and can be captured and stabilized by FAs (Small et al., 2002; Stehbens and Wittmann, 2012; Etienne-Manneville, 2013). Accordingly, we observed a co-occurrence of transport tracks with the microtubule network (Fig. 2 G), suggesting that a subset of microtubules targeting FAs is used to release cargos. Kymographs drawn along the tracks (in yellow) indicated that the same tracks are used several times by distinct transport carriers coming from the Golgi apparatus in the direction of the plasma membrane (Fig. 2 H). While microtubules are abundant in cells, transport carriers thus seem to select microtubule subsets (Fig. 2, F–H). Kymographs and line scans also illustrated that secretion of cargo does not occur on FAs but very close to them, in agreement with TIRF experiments (Fig. 2 H). This may be explained by steric hindrance due to the abundance and tight arrangement of proteins at FAs and/or to specific localization of targeting/fusion factors. Altogether, the above results indicate that secretory vesicles use preferential and direct microtubule-based routes for transport to secretion hotspots.

### ELKS- and RAB6-dependent arrival of secreted cargos at exocytosis hotspots

**Figure 3.**
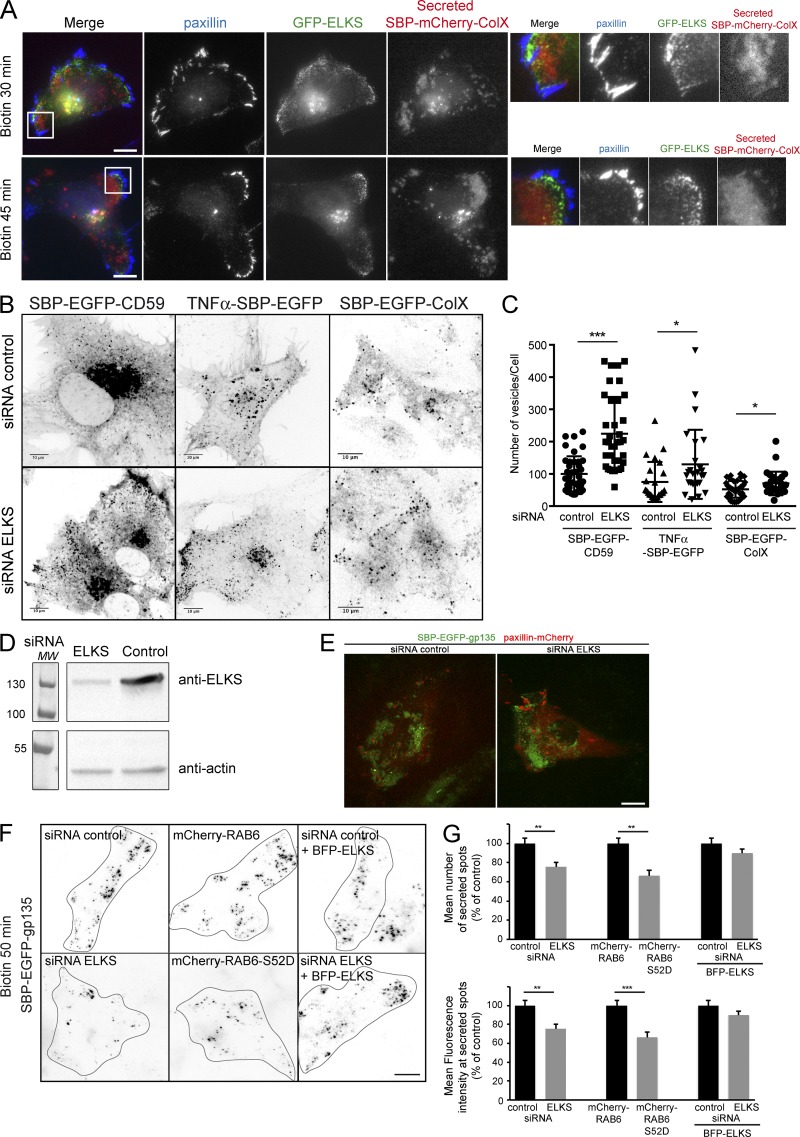
**ELKS- and RAB6-dependent arrival of secreted cargos at exocytosis hotspots. (A)** HeLa cells were transfected with SBP-mCherry-ColX and GFP-ELKS. After 30- or 45-min incubation with biotin, cells were processed for immunofluorescence and stained with an anti-paxillin antibody. Coverslips were coated with an anti-GFP antibody (SPI assay). Higher magnifications of the images are shown on the right. **(B)** HeLa cells expressing SBP-EGFP-CD59, TNFα-SBP-EGFP, and SBP-EGFP-ColX were treated for 3 d with control or ELKS siRNAs. Cells were incubated for 60–90 min with biotin to allow cargo release from the ER and its arrival to the plasma membrane. Representative images taken from videos. **(C)** The number of vesicles per cell were quantified using ImageJ (mean ± SEM, *n* = 23–39 cells). *, P < 0.05; ***, P < 10^−4^ (Student’s *t* test). Cells were treated as indicated in B. Scale bars, 10 µm. **(D)** HeLa cells were treated with control or ELKS siRNA and processed for Western blotting. ELKS signal was revealed using an anti-ELKS antibody. Actin signal was used as a loading control. **(E)** HeLa cells were treated with control or ELKS siRNA and then cotransfected with SBP-EGFP-gp135 and paxillin-mCherry. Cells were seeded on anti-GFP–coated coverslips (SPI), and trafficking of SBP-EGFP-gp135 was monitored by spinning disk microscope upon addition of biotin. **(F)** HeLa cells were treated with control or ELKS siRNA and then transfected with SBP-EGFP-gp135. For rescue experiments, cells were cotransfected with BFP-ELKS. Cells were seeded on anti-GFP–coated coverslips (SPI). After a 50-min treatment with biotin, cells were fixed. Representative images are displayed. **(G)** The number of secreted spots as well as their intensity was quantified using ImageJ or Fiji software (mean ± SEM, *n* = 39–60 cells). Cells were treated as indicated in F. **, P < 10^−2^; ***, P < 10^−4^ (Student’s *t* test). Scale bars, 10 µm.

Microtubules are attached to the cell cortex via the microtubule-stabilizing proteins CLASPs, which interact with a protein complex made of LL5β and ELKS (Lansbergen et al., 2006). We therefore investigated the presence of ELKS in secretion hotspots. As shown in Fig. 3 A, GFP-ELKS was enriched in zones of the plasma membrane close to FAs, where immobilized ColX is detected 30–45 min after biotin addition.

ELKS is a RAB6 effector (Monier et al., 2002) and was shown to be involved in the docking of RAB6-positive secretory vesicles containing VSV-G and NPY to the plasma membrane (Grigoriev et al., 2007). In ELKS-depleted cells, in agreement with data reported previously (Grigoriev et al., 2007), we observed an accumulation of SBP-EGFP-CD59–, TNFα-SBP-EGFP–, or SBP-EGFP-ColX–positive vesicles at the cell periphery as well as an increase in the total number of cytoplasmic vesicles (Fig. 3, B and C). As previously shown (Lansbergen et al., 2006), we verified that ELKS depletion has a small but significant impact on the size of FAs (Fig. S2).

In cells depleted for ELKS by siRNAs, the secretion was still biased toward the regions near a subset of FA (Fig. 3, D and E). Quantification showed that the number of secreted spots as well as their intensity was decreased (Fig. 3, F and G). We then evaluated the role of the direct RAB6-ELKS interaction in this process. We recently identified a RAB6 mutant, RAB6-S52D, that does not interact with ELKS (unpublished data and Fig. S3, A and B). In an attempt to outcompete endogenous RAB6, we overexpressed GFP-RAB6-S52D and observed that it did result in displacement of ELKS (Fig. S3 C). In addition, GFP-RAB6-S52D was found on post-Golgi vesicles (Fig. S3 D). Next, we monitored the effect of expression of mCherry-RAB6-S52D on the transport of SBP-EGFP-gp135 and observed that it phenocopies ELKS depletion: secreted spots of cargo decreased in the number and intensity. In addition, secretion was still biased toward FAs (Fig. 3, F and G). Altogether, these data show that RAB6 and ELKS play a role in the docking and fusion of cargo-containing secretory vesicles with the plasma membrane.

### RAB6 associates with post-Golgi carriers containing GPI-APs, TNFα, and ColX

**Figure 4.**
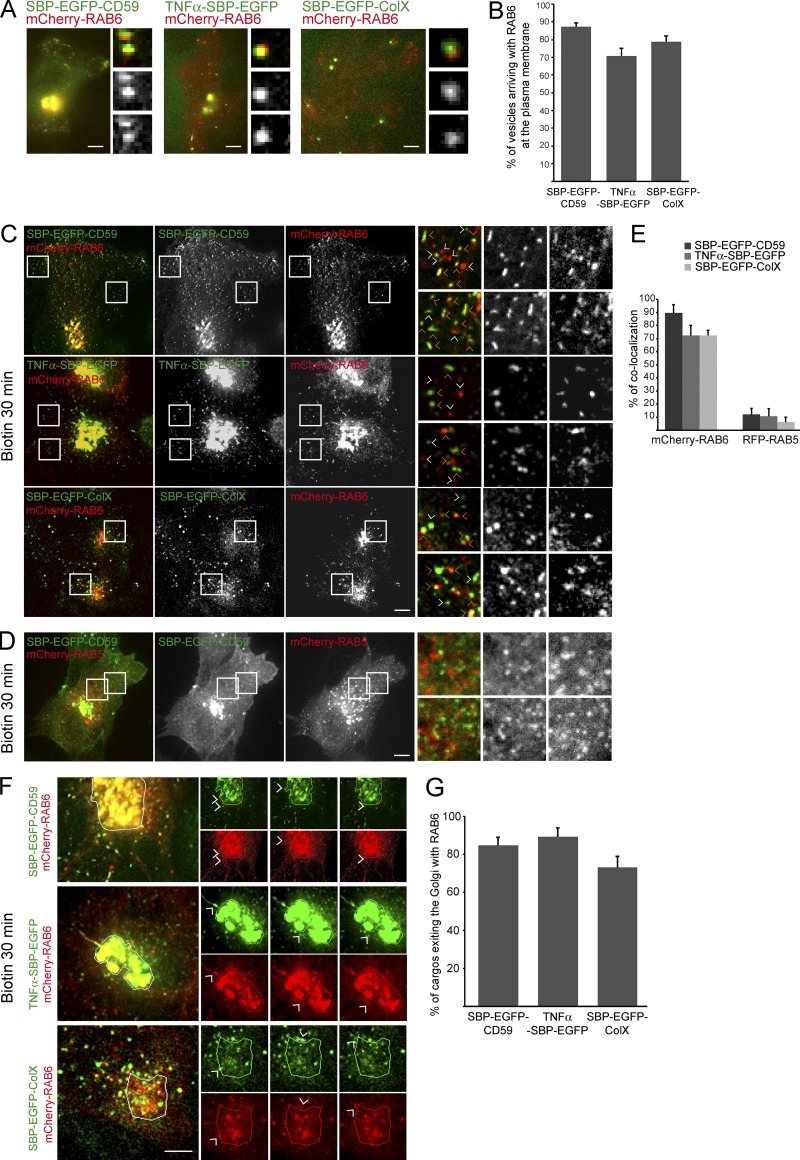
**RAB6 associates with post-Golgi carriers containing CD59, TNFα, or ColX. (A)** HeLa cells coexpressing mCherry-RAB6 together with SBP-EGFP-CD59, TNFα-SBP-EGFP, and SBP-EGFP-ColX were incubated for 45 min with biotin and imaged using 3D-TIRF. Representative images taken from the videos are displayed. **(B)** Quantification of the percentage of RAB6-positive vesicles arriving at the plasma membrane and containing SBP-EGFP-CD59, TNFα-SBP-EGFP, or SBP-EGFP-ColX. Cells were treated as indicated in A (mean ± SEM, *n* = 217–325 vesicles from 14–18 cells). **(C)** RPE1-SBP-EGFP-CD59, HeLa-SBP-EGFP-ColX (stably expressing cells or transiently transfected cells), and HeLa-TNFα-SBP-EGFP cells coexpressing mCherry-RAB6 were incubated for 30 min with biotin to allow cargo release from the ER. Cells were imaged using a time-lapse spinning-disk confocal microscope. Representative images taken from videos are displayed. Orange arrowheads point at colocalized vesicles. White arrowheads point at non-colocalized vesicles. **(D)** HeLa cells coexpressing SBP-EGFP-CD59 and RFP-RAB5A were incubated for 30 min with biotin to allow cargo release from the ER. Cells were imaged using a time-lapse spinning-disk confocal microscope. Representative images taken from videos are displayed. **(E)** Quantification of the colocalization between mCherry-RAB6 or m-Cherry-RAB5 and each type of cargo (mean ± SEM, *n* = 9–24 cells). (**F)** HeLa cells coexpressing mCherry-RAB6 and SBP-EGFP-CD59, TNFα-SBP-EGFP, or SBP-EGFP-ColX were incubated for 15–20 min with biotin and imaged using time-lapse video-microscopy. Representative images of vesicles positive for SBP-EGFP-CD59, TNFα-SBP-EGFP, or SBP-EGFP-ColX and mCherry-RAB6 exiting the Golgi complex together are displayed. Higher magnification of the images taken from time-lapse videos are on the right. **(G)** Quantification of the percentage of EGFP-SBP-CD59–positive vesicles exiting the Golgi complex with RAB6 (mean ± SEM, *n* = 13–18 cells). Scale bars, 10 µm.

The very first study on the dynamics of GFP-RAB6 in living cells reported the presence of peripheral RAB6-positive structures near FAs (White et al., 1999). We thus used TIRF microscopy to test whether RAB6 was also associated with the transport carriers containing the anterograde cargos tested in this study (GFP-tagged ColX, TNFα, or CD59). We observed that ∼80% of vesicles arriving at the plasma membrane and containing one of these cargos were positive for RAB6 (Fig. 4, A and B). We next investigated whether RAB6 was associated with post-Golgi carriers containing CD59, TNFα, or ColX en route to the plasma membrane. Using live-cell imaging, we performed a detailed analysis of the extent of colocalization between mCherry-RAB6 and several cargos upon Golgi exit (SBP-EGFP-CD59, TNFα-SBP-EGFP, or SBP-EGFP-ColX). Following a 30-min incubation with biotin, we observed that 80% of vesicles containing one of the cargos were RAB6-positive (Fig. 4, C and E). By contrast, only 2–5% of RAB5-positive endosomal vesicles, used here as a negative control, were positive for CD59, ColX, or TNFα in these conditions (Fig. 4, D and E). As expected, colocalization between RAB6 and cargos was minimal (10%) in post-ER compartments reached 10 min after biotin addition (Fig. S4).

To rule out the possibility that the high percentage of RAB6-positive post-Golgi transport carriers is due to mCherry-RAB6 overexpression, the anti-RAB6:GTP antibody AA2 (Nizak et al., 2003) was used to analyze endogenous RAB6 expression. 60% colocalization was obtained between endogenous RAB6:GTP and SBP-EGFP-CD59, TNFα-SBP-EGFP, or SBP-EGFP-ColX present in post-Golgi vesicles (Fig. S4), indicating that the high percentage of post-Golgi transport carriers positive for RAB6 was not a consequence of RAB6 overexpression.

To investigate when RAB6 associates with post-Golgi carriers, we carefully examined them at the exit of the Golgi. As shown in Fig. 4, F and G, ∼80% of vesicles containing SBP-EGFP-CD59, TNFα-SBP-EGFP, or SBP-EGFP-ColX exiting the Golgi complex 30 min after biotin addition were positive for RAB6. Importantly, this percentage of colocalization is similar to the 80% colocalization we found above when looking at the whole population of transport carriers. Altogether, our results show that RAB6 is present on transport vesicles, irrespective of the transported cargo, when they leave the Golgi and remains associated with them until they reach the plasma membrane.

### The RAB6 machinery is required for the secretion of GPI-APs, TNFα, and ColX

**Figure 5.**
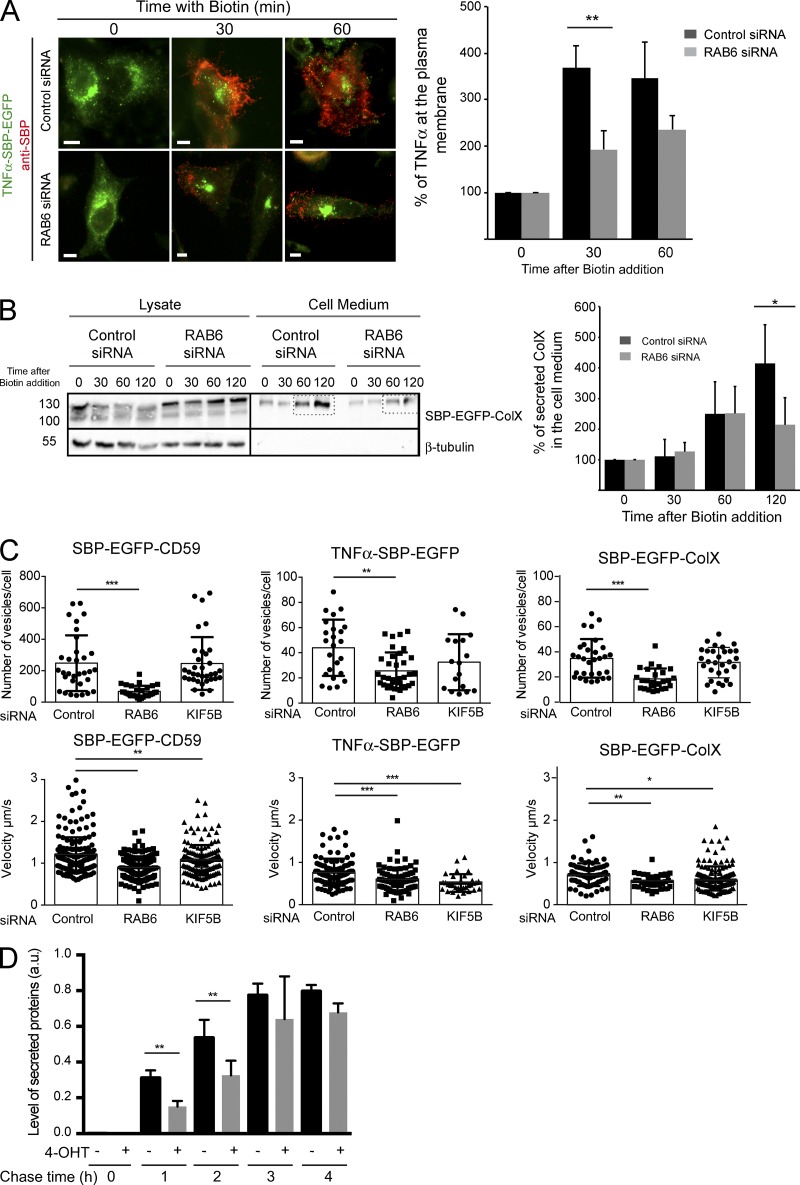
**RAB6 and KIF5B are required for the proper secretion SBP-EGFP-CD59, TNFα-SBP-EGFP, and SBP-EGFP-ColX at the plasma membrane; RAB6 depletion delays total protein secretion. (A)** Left: HeLa-TNFα-SBP-EGFP, treated or not with RAB6 siRNAs, were incubated for 30 or 60 min with biotin to allow cargo release. The amount of secreted TNFα was determined on images acquired on cells using the SPI assay. Representative images of cells expressing TNFα-SBP-EGFP (green) and stained with anti-SBP (red) are displayed. Right: Quantification of the amount of TNFα present at the plasma membrane in cells treated as above (mean ± SEM, *n* = 67–111 cells). **, P < 10^−2^ (Student’s *t* test). Scale bars, 5 µm. **(B)** Left: HeLa-SBP-EGFP-ColX treated or not with RAB6 siRNAs were incubated for 30, 60, or 120 min with biotin to allow cargo release from the ER. The amount of secreted cargo was revealed by Western blotting using an anti-GFP antibody. β-tubulin was used as a loading control and as a nonsecreted protein. Representative immunoblots are displayed. Right: Quantification of the amount of ColX present at the plasma membrane in cells treated as indicated above (mean ± SEM, *n* = 3). *, P < 0.05 (Student’s *t* test). **(C)** HeLa cells expressing SBP-EGFP-CD59, TNFα-SBP-EGFP, and SBP-EGFP-ColX were treated for 3 d with control, RAB6, or KIF5B siRNAs. Cells were incubated for 30 min with biotin to allow cargo release from the ER. Cells were imaged using a time-lapse spinning disk confocal microscope. Vesicle velocity (µm/s) and the number of vesicles per cell were quantified using ImageJ (mean ± SEM, *n* = 17–35 cells). *, P < 0.05; **, P < 0.005; ***, P < 10^−4^ (Student’s *t* test). **(D)** The SUnSET assay was used to determine the effect of RAB6 depletion on total protein secretion. MEFs prepared from RAB6 loxP/Ko Rosa26CreERT2-TG embryos (described in Bardin et al. [2015]; named after MEF RAB6^lox/lox^) were treated with ethanol or with 4-OHT for 96 h to induce RAB6 depletion. Cells were then incubated with puromycin and chased in puromycin-free medium for 0, 1, 2, 4, or 5.5 h. Total protein content in cell lysis or supernatant was labeled with an anti-puromycin antibody. Puromycin intensity in the supernatant and the whole cell lysis was quantified using Image Lab software (Bio-Rad; mean ± SEM, *n* = 4). **, P < 0.05 (Student’s *t* test). Representative immunoblot is displayed in Fig. S5. a.u., arbitrary units.

To address the functional role of RAB6, we assessed the effect of siRNA-mediated knockdown of RAB6 on the secretion of various cargos. A 50% inhibition of the arrival of TNFα at the plasma membrane was observed 30 and 60 min after biotin addition under conditions of RAB6 depletion, as quantified using SPI (Fig. 5 A). Similarly, in the absence of RAB6, a 50% reduction of ColX secretion was observed 120 min after biotin addition as analyzed by Western blotting of cell lysates and culture medium (Fig. 5 B). The arrival at the plasma membrane of PLAP was also delayed (Fig. S5 A). RAB6 has been shown to cooperate with RAB8 in post-Golgi trafficking (Grigoriev et al., 2007). RAB8 was found to be colocalized with RAB6 on post-Golgi carriers, and codepletion of RAB6 and RAB8 did not lead to additional effects on the transport of PLAP (Fig. S6), indicating that RAB6 and RAB8 act in the same post-Golgi trafficking pathway. In contrast and as expected, RAB11 was not found associated with post-Golgi vesicles (Fig. S6).

RAB6 depletion leads to a 50% decrease in the number of post-Golgi vesicles containing VSV-G (Grigoriev et al., 2007; Miserey-Lenkei et al., 2010), which is similar to the reduction we observed in the number of SBP-EGFP-CD59–, TNFα-SBP-EGFP–, or SBP-EGFP-ColX–positive post-Golgi vesicles (Fig. 5 C). We have shown before that this reduction results from a decreased recruitment on Golgi/TGN membranes of myosin II, whose activity is required for the fission of RAB6-positive vesicles from Golgi/TGN membranes (Miserey-Lenkei et al., 2010). To confirm the involvement of myosin II in the transport of the RUSH cargos, cells expressing SBP-EGFP-CD59, TNFα-SBP-EGFP, or SBP-EGFP-ColX were incubated with the myosin II inhibitor para-nitroblebbistatin for 15 min before adding biotin for 30 min. As illustrated in Fig. S5 (B and C), SBP-EGFP-CD59 could be detected in membrane tubules connected to the Golgi that represent transport carriers that cannot detach from Golgi/TGN membranes (Miserey-Lenkei et al., 2010).

Finally, RAB6-positive vesicles are transported from the Golgi to the plasma membrane along microtubules by the kinesin family member 5B (KIF5B) kinesin motor (Grigoriev et al., 2007; Miserey-Lenkei et al., 2010). Accordingly, cells treated with KIF5B siRNA showed a reduced velocity of CD59-, TNFα-, or ColX-positive post-Golgi vesicles (Fig. 5 C). Of note, RAB6 or KIF5B depletion does not affect the size of FAs (Fig. S2).

Altogether, the above results show that a variety of cargos use the RAB6 machinery for transport from Golgi to the plasma membrane. They also raise the possibility that RAB6 might be involved in the secretory process of all proteins leaving the Golgi complex. To test this hypothesis, we performed experiments on mouse embryonic fibroblast (MEF) cells derived from *RAB6* conditional knockout (KO) mice embryos whose *RAB6* alleles can be mutated using 4-hydroxytamoxifen (4-OHT) to activate the Cre recombinase (MEF RAB6^lox/lox^, Bardin et al., 2015). Global protein secretion was monitored using the Surface Sensing of Translation (SUnSET) assay (Schmidt et al., 2009). MEFs RAB6^lox/lox^ treated with either 4-OHT or with ethanol as a control (Fig. S5) were incubated with low concentrations of puromycin and then chased in puromycin-free medium for different times. The amount of protein in cell lysates and supernatants was analyzed by Western blot using an anti-puromycin antibody, and the relative amount of secreted proteins was quantified (Figs. 5 D and S5). To assess that the SUnSET assay worked properly, the same experiments were performed following incubation with the protein synthesis inhibitor cycloheximide, and as expected, no labeled proteins were detected in both cell lysates and supernatants (Fig. S5). Following RAB6 depletion, the total amount of secreted protein after a 1- or 2-h chase was reduced by 50%, although total protein expression was unaffected (see cell extracts at *t* = 0 of cell treated or not with 4-OHT, Fig. S5). As apparent on the Western blots, the intensity of all secreted protein bands was decreased upon RAB6 depletion, suggesting a global effect on protein secretion (Figs. 5 D and S5). This effect was rescued by overexpressing mCherry-RAB6 (Fig. S5). In contrast, expression of the RAB6 mutant mCherry-RAB6-S52D, which cannot interact with ELKS, could not rescue the inhibition of secretion (Fig. S5). Importantly, after 4 and 5.5 h of chase, the amount of protein secreted by cells expressing RAB6 or not was similar (Fig. 5 D). These results thus showed that RAB6 depletion does not block the secretory process but leads to a delay in total protein secretion, as previously found for exogenous cargos (Grigoriev et al., 2007; Miserey-Lenkei et al., 2010). Altogether, the above results show that RAB6 function is required for total protein secretion at the cell level.

### RAB6 is not involved in sorting of cargos at the exit of Golgi complex

**Figure 6.**
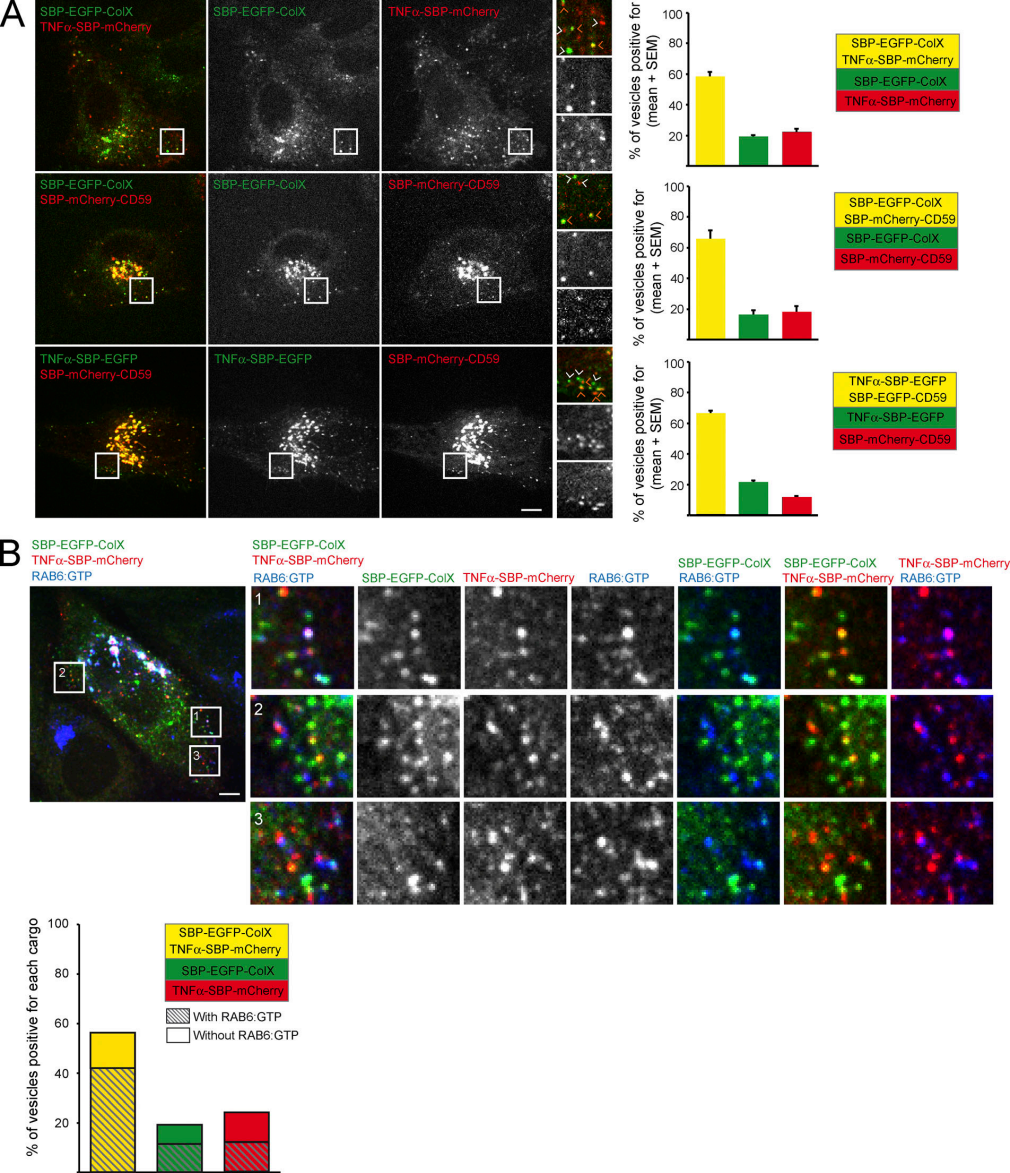
**RAB6 is not involved in sorting of cargos at the exit of Golgi complex. (A)** HeLa cells coexpressing SBP-EGFP-ColX and TNFα-SPB-mCherry, SBP-EGFP-ColX and SBP-mCherry-CD59, or TNFα-SBP-EGFP and SBP-mCherry-CD59 were incubated for 35 min with biotin to allow cargo release from the ER and then imaged using time-lapse video microscopy. Representative images taken from videos are shown (higher magnification on the right). Orange arrowheads point at colocalized vesicles; white arrowheads point at non-colocalized vesicles. Right: Quantification for each pair of cargos of the percentage of vesicles positive both cargos (yellow), EGFP (green), or mCherry (red; mean ± SEM, *n* = 197–501 vesicles from 11–20 cells). **(B)** Staining of endogenous RAB6:GTP (blue) in cells coexpressing SBP-EGFP-ColX (green) and TNFα-SBP-mCherry (red) and incubated for 30 min with biotin to visualize post-Golgi carriers. Higher magnifications are shown on the right. Quantification of the percentage of vesicles containing both SBP-EGFP-ColX and TNFα-SPB-mCherry (yellow), only SBP-EGFP-ColX (green), or only TNFα-SPB-mCherry (red). Dashed bars indicate the percentage of vesicles positive for RAB6 (mean ± SEM, *n* = 24–128 vesicles). Scale bars, 5 µm.

Newly synthesized proteins are thought to be sorted into distinct populations of transport carriers at the TGN (De Matteis and Luini, 2008). The RUSH system allows synchronization of transport of two (or more) cargos at the same time, thus providing a powerful approach to investigate sorting processes in further detail. We imaged cells expressing two cargos, SBP-EGFP-ColX and TNFα-SBP-mCherry, SBP-EGFP-ColX and SBP-mCherry-CD59, or TNFα-SBP-EGFP and SBP-mCherry-CD59 (Fig. 6 A). In all cases, while a majority (60%) of the vesicles contained two cargos, a large fraction of vesicles contained only one cargo. This indicates that, despite the sudden wave of transport imposed by biotin-induced release from the ER, efficient sorting still occurs at the Golgi complex. We then estimated the percentage of colocalization between RAB6 and post-Golgi vesicles containing one or two cargos. As illustrated in Fig. 6 B in the case of TNFα and ColX, the majority (60%) of vesicles containing the two cargos were positive for endogenous RAB6. Importantly, a similar percentage of vesicles containing only one cargo were positive for RAB6 (50% and 60% of vesicles containing ColX or TNFα, respectively; Fig. 6 B). This suggests that RAB6 is not involved in sorting of cargos at the exit of the Golgi complex but associates with transport carriers, irrespective of the transported cargo, to target them to specific sites at the plasma membrane.

## Discussion

Cells need to continuously control the transport of various proteins to the cell surface both to ensure homeostasis and to sustain differentiated functions. The study of various transport steps, such as ER to Golgi or Golgi to ER, have shown that mechanisms are at work that enable the transport of a diversity of proteins using a “universal” core machinery (coat protein complex I [COPI] and coat protein complex II [COPII], for example). However, whether Golgi to plasma membrane is similarly controlled by a core machinery was still unclear. One of the main findings of this study is that post-Golgi transport of diverse secretory cargos, of various shapes and functions and characterized by diverse transport kinetics, are handled by a common machinery and reach the membrane in similarly restricted domains.

The concept of “exocytosis hotspots” existing in cells was actually proposed almost 40 yr ago (Louvard, 1980) when analyzing the secretion of fibronectin after cell attachment (Heggeness et al., 1978). Surprisingly, we found that the secretion of cargos recently released from the Golgi complex occurs in so-called hotspots close to FAs and that this is a general process common to several and diverse proteins.

Targeting to FAs does not seem to depend on the particular function or modification of the cargo, because it was observed for *N*-glycosylated (gp135 and ColX) and *O*-glycosylated proteins (TNFα). This was also observed for soluble secretory GFP, which is nonglycosylated and unlikely to bear any specific transport signal. The secretion of particular cargo types, such as matrix metalloproteinases (e.g., membrane-type matrix metalloproteinase 1 [MT1-MMP]), at adhesion sites was previously proposed (Bravo-Cordero et al., 2007; Stehbens et al., 2014). However, it was observed only for MT1-MMP, and it was not clear if the vesicles containing MT1-MMP were strictly secretory vesicles or vesicles recycled from the endocytic/recycling pathways. Here, by synchronizing the anterograde transport of several cargos using the RUSH assay, we unambiguously analyzed the first wave of secretion of transport carriers. The SPI assay that we set up for capture of secreted proteins prevented transmembrane diffusion in the plasma membrane or release of soluble cargos into the extracellular space. The SPI provides insight into the secretion history of synchronized cargos. Of note, the SPI assay allows the capture of secretion events that occur at the ventral surface only, and in the future it will be important to analyze transport in a 3D environment. By combining the RUSH and SPI setups, we were able to show that transport carriers use preferential microtubule tracks to reach secretion hotspots. In addition to this directed movement, the exocytic zones may be restricted close to FAs by the presence of factors necessary for docking/fusion of transport carriers such as ELKS and LL5β proteins (Grigoriev et al., 2011). Recently, a role for the Rho signaling pathway in the local delivery of RAB6 vesicles at FAs was described (Eisler et al., 2018). In addition, the αTAT1 acetylase was shown to be involved in the fusion of RAB6-positive vesicles at FAs (Bance et al., 2019).

Focal adhesions both produce and sense adhesion forces. They are able to modulate cell adhesion in response to cellular environment, for instance by modifying attachment to the extracellular matrix. At the intracellular level, the interaction between microtubules and FAs impacts the structure and dynamics of FAs. This interaction is essential for cellular organization, polarization, and migration (van der Vaart et al., 2009). Here we observed that their localization is correlated to the existence of secretion hotspots. Since we could not observe the early steps of FA establishment while monitoring exocytosis, it is not possible to precisely decipher the mechanisms allowing FAs and exocytosis to occur in these domains. Focal adhesions could dictate the presence of secretion hotspots, restricted exocytosis domains could favor FAs, or upstream mechanisms such as local absence of cortical actin or the accumulation of adhesion proteins in plasma membrane subdomains could enable FAs and exocytosis to occur in these hotspots.

**Figure 7.**
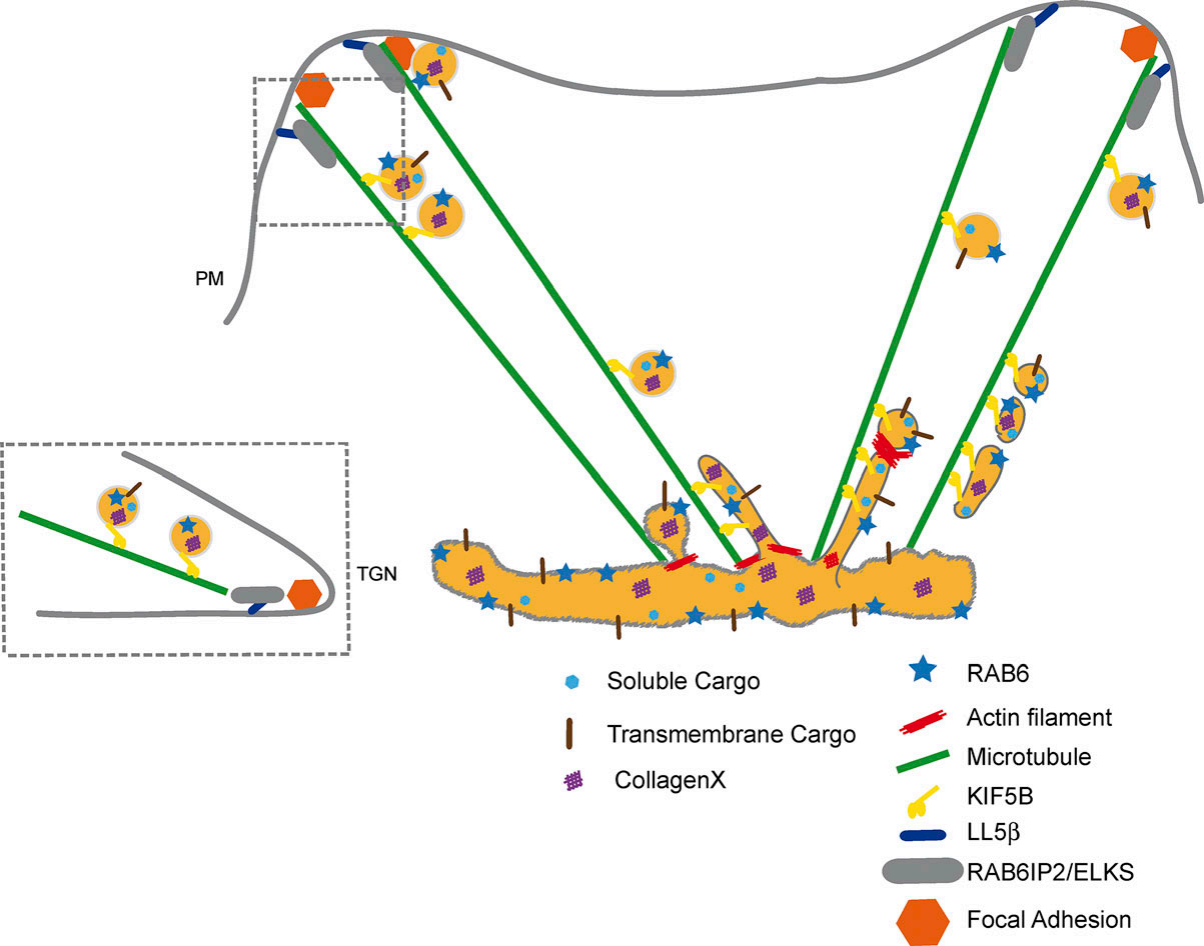
**Proposed model to explain the role of RAB6 in post-Golgi secretory pathway.** All RAB6 effectors associated to post-Golgi secretion, myosin II, KIF5B, and ELKS/RAB6IP2, are involved in the secretion of various cargos. The different events can be envisioned as follows. RAB6 is present on CD59-, TNFα-, and ColX-containing vesicles exiting the Golgi complex. Myosin II is implicated in the fission of these vesicles from Golgi membranes (Miserey-Lenkei et al., 2010). Vesicles are transported to the plasma membrane in a KIF5B-dependent manner (Grigoriev et al., 2007; Miserey-Lenkei et al., 2010), and ELKS ensures their docking to exocytic hotspots close to FAs (Grigoriev et al., 2007, 2011).

An important finding of this study is that RAB6 is likely a major regulator of post-Golgi secretion (Fig. 7). RAB6 was previously shown to be present on post-Golgi transport carriers containing VSV-G, NPY, and TNFα (Grigoriev et al., 2007; Miserey-Lenkei et al., 2010; Micaroni et al., 2013; Johns et al., 2014). Here we show that RAB6 associates with secretory vesicles containing a variety of exogenous cargos and that its depletion delays their arrival at the plasma membrane. The observation that the secretion of most endogenous proteins synthesized by MEF cells is affected by RAB6 depletion further suggests that RAB6 may be present on the majority of post-Golgi vesicles transporting cargos to the plasma membrane. This is in agreement with a model proposing that one of the main functions of RAB6 is to target post-Golgi vesicles to cortical ELKS-containing patches that define secretion domains (Grigoriev et al., 2007, 2011). In our study, we show that ELKS and RAB6 are already associated on the post-Golgi vesicles en route to the plasma membrane and that RAB6 is necessary to load ELKS on these vesicles. In addition, ELKS may dock RAB6-positive vesicles not only to the plasma membrane but also to other compartments. We have recently shown that ELKS is present on the membrane of mature melanosomes in melanocytes, which allows the diversion of part of the RAB6-dependent secretory pathway to melanosomes, enabling direct transport of a subset of melanosomal enzymes from the Golgi (Patwardhan et al., 2017).

We have previously shown that RAB6 does not only control the fission of its own transport carriers (Miserey-Lenkei et al., 2010) but also could select a subset of microtubules originating from Golgi membranes for their transport (Miserey-Lenkei et al., 2017). Although not directly proven, it is likely that this subset of microtubules correspond to the ones identified in this study, which are used several times for targeting cargos to secretion hotspots.

Several findings suggest that multiple classes of secretory carriers can form at the trans-Golgi, indicating that cargos are sorted before being delivered to the plasma membrane (De Matteis and Luini, 2008). Our study highlights that, even when pulsing a massive amount of cargos in the secretory pathway, the Golgi is still able to properly sort a large percentage of cargos in dedicated transport intermediates. Such sorting may be even more important in polarized cells, such as epithelial or neuronal cells, or when studying cargos targeted to particular intracellular compartments such as the endolysosomal system. Intra-Golgi sorting is also evident when monitoring the time of residence in the Golgi and exit from the Golgi of cargo. Some cargos exit very quickly, while others stay for a long time in the Golgi complex, suggesting intra-Golgi segregation. However, our data indicate that irrespective of the cargo, the vast majority of transport intermediates are positive for RAB6. This suggests that RAB6 is not involved in cargo sorting but belongs to a general machinery responsible for fission of transport intermediates from the Golgi (Miserey-Lenkei et al., 2010) and their fusion with target compartments.

In conclusion, our study indicates that despite the diversity of Golgi-intersecting routes, most post-Golgi transport is controlled by a RAB6-dependent core machinery that drives secretion on exocytic hotspots close to FAs. Why localized exocytosis domains exist remains an open question and will require further work, in particular in 3D environment and tissues. These domains may simply reflect the global architecture of the cell dictated by the organization of the actin and microtubule networks. Alternatively, they may result from functional links existing between Golgi membranes and FAs, which are important for cell adhesion, polarization, and migration.

## Materials and methods

### Cells

HeLa and RPE-1 cells were grown at 37°C with 5% CO_2_ in DMEM (high glucose; GlutaMAX; Life Technologies) or DMEM-F12 (Gibco), respectively, supplemented with 10% FCS (GE Healthcare and Eurobio), 1 mM pyruvate sodium (Life Technologies), and 100 U/ml penicillin and streptomycin (Life Technologies and Gibco). HeLa cells stably expressing RUSH constructs were cultured as described previously (Fourriere et al., 2016) in medium supplemented with 4 µg/ml of puromycin (Invitrogen). Lentiviral infection was used to generate RPE-1 cells stably expressing streptavidin-KDEL and SBP-EGFP-CD59. MEFs were prepared from mouse *RAB6* loxP/loxP (control) or *RAB6* loxP/KO Rosa26CreERT2-TG (MEF RAB6^lox/lox^) mouse embryos (Bardin et al., 2015). MEF cells were grown in DMEM (Gibco) supplemented with 10% FCS (Eurobio) and 100 U/ml penicillin/streptomycin (Gibco). For RAB6 depletion, MEF cells were incubated with 1 µM 4-OHT for 96 h.

### Plasmids

We used the plasmids encoding the following fusion proteins: GFP-/mCherry-RAB6A or -RAB6A′ (Miserey-Lenkei et al., 2010); GFP-ELKS (a gift from A. Akhmanova, Utrecht University, Utrecht, The Netherlands); and VSV-GtsO45-EGFP (Hirschberg et al., 1998). Paxillin-BFP, paxillin-GFP, and paxillin-mCherry were constructed from the human paxillin cDNA from Open Biosystems (accession no. BC144410); GFP-/mCherry-RAB8A (a gift from Z.L. Zhao, Mechanobiology Institute, Singapore), mCherry-RAB11A (a gift from J. Salamero, Institut Curie, Paris, France). All the RUSH plasmids used in this study (except VSV-G-SBP-EGFP) use streptavidin-KDEL as a hook. VSV-G-SBP-EGFP uses streptavidin-Ii as a hook. Briefly, the hook (streptavidin-tagged protein) allows anchoring of the SBP-tagged reporter in the ER in the absence of biotin thanks to streptavidin–SBP interaction. RUSH plasmids coding for TNFα-SBP-EGFP, TNFα-SBP-mCherry, TNFα-SBP-tagBFP, SBP-EGFP-E-cadherin, and SBP-ssEGFP were previously described (Boncompain et al., 2012; Fourriere et al., 2016). The other RUSH plasmids used were generated as previously described (Boncompain and Perez, 2013). The accession numbers of the corresponding reporter used as template are as follows: ColX (BC130621), gp135/podocalyxin (NP_001076235), CD59 (NP_000602), and PLAP (NP_001623). The release of the RUSH cargos was induced by addition of 40 µM of d-biotin (Sigma-Aldrich). GFP- and mCherry-tagged RAB6A-S52D were constructed as follows: a single point mutation was inserted in the human RAB6A sequence at position 156 using the QuikChange mutagenesis kit (Agilent). The rescue plasmid coding for FK506 binding protein (FKBP)-tagBFP-ELKS full length bears a FKBP-rapamycin–binding (FRB) domain fused to a plasma membrane anchor (from Lyn) upstream of a T2A sequence. PM-FRB-T2A_FKBP-tagBFP sequence flanked with NheI and BamHI was ordered as synthetic DNA from Integrated DNA Technologies. The synthetic DNA was cloned in the plasmid PM-FRB-mRFP-T2A-FKBP-5-ptase, which was a gift from Peter Varnai (Semmelweis Univeristy, Budapest, Hungary; Addgene plasmid 40896; RRID: Addgene_40896). ELKS full length was inserted from EGFP-ELKS plasmid (kind gift from A. Akhmanova) using EcoRI and NotI restriction sites.

### Biochemical reagents

The following reagents were used: para-nitroblebbistatin (Optopharma), 4-OH (Sigma-Aldrich), and d-biotin (Sigma-Aldrich). SiR-tubulin (Spirochrome) was used to label microtubules in living cells according to the manufacturer’s instructions.

### DNA and RNA transfection

HeLa or RPE-1 cells were transfected 24–48 h before observation with calcium phosphate (Jordan et al., 1996) or with X-tremeGEN9 (Roche), following the manufacturer’s instructions. For rescue experiments in MEF cells (Fig. S5), mCherry-RAB6wt or mCherry-RAB6-S52D were transfected using Lipofectamine 3000 (Invitrogen), following the manufacturer’s instructions. For RNA interference experiments, cells were transfected with the corresponding siRNA (RAB6A/A′, KIF5B, ELKS, RAB8A, or Luciferase) using Lipofectamine RNAiMAX (Invitrogen) or Hiperfect (Qiagen), following the manufacturer’s instructions. The sequences of the siRNAs used in this study are as follows: siRNA Luciferase (5′-CGU​ACG​CGG​AAU​ACU​UCG​A-3′, Sigma-Aldrich); siRNA against human RAB6 targeting RAB6A/A′ (Del Nery et al., 2006; 5′-GAC​AUC​UUU​GAU​CAC​CAG​A-3′, Sigma-Aldrich); siRNA against human KIF5B (Grigoriev et al., 2007; 5′-AAC​GTT​GCA​AGC​AGT​TAG​AAA-3′, Sigma-Aldrich); siRNA against human RAB8A (Grigoriev et al., 2011; 5′-GGA​AAG​CAC​AAA​UGA​AGG​A-3′, Sigma-Aldrich); and siRNA against ELKS (Grigoriev et al., 2007; 5′-GUG​GGA​AAA​CCC​UUU​CAA​U-3′, Ambion).

### Yeast two-hybrid experiments

Yeast two-hybrid experiments were performed as described in Echard et al. (1998) except that LexA-fusion proteins corresponded to ELKS full length described in Monier et al. (2002). Briefly, the *Saccharomyces cerevisiae* reporter strain L40 was cotransformed with a plasmid encoding GAL4 fusion proteins to detect interactions between domain of full-length ELKS and RAB6A-Q72L or RAB6A-Q72L-S52D. Cells were grown on medium containing (-L-W) or lacking (-L-W-H) histidine; growth on medium without histidine indicates an interaction between the encoded proteins.

### SPI assay

Coverslips were incubated in sterile conditions in a bicarbonate solution (0.1 M, pH 9.5, 1 h, 37°C) followed by incubation in 0.01% poly-l-lysine (diluted in water, 1 h, 37°C; Sigma-Aldrich). Coverslips were then washed in PBS and dried before antibody incubation (diluted in the bicarbonate buffer) for 3 h at 37°C (or overnight). Coverslips were washed twice in PBS and then with cell medium before cells were seeded. Antibodies used for coating in this study were rabbit anti-GFP (A-P-R#06; Recombinant Antibody Platform of the Institut Curie; dilution 1:50 to 1:400), rabbit anti-mCherry (A-P-R#13; Recombinant Antibody Platform, Institut Curie; dilution 1:400), mouse anti-VSV-G (a gift from T. Kreis, University of Geneva, Switzerland; dilution 1:50), and mouse anti-SBP (Millipore; MAB10764, batch 2697549, dilution 1:60). To detect the coated antibodies, either donkey anti-rabbit or donkey anti-mouse antibodies conjugated to Cy3 (Jackson Immunoresearch) were used. To test specific binding to coated antibodies, recombinant GFP (Recombinant Protein Platform, Institut Curie) was incubated for 40 min at dilutions indicated in Fig. S1.

### Immunofluorescence

Cell fixation was performed with 3% or 4% PFA (Electron Microscopy Sciences) for 15 min at room temperature. For permeabilization, cells were incubated in PBS supplemented with 2 g/liter BSA and 0.5 g/liter saponin for 10 min at room temperature. Surface staining was performed at 4°C on nonfixed intact cells incubated with the primary antibody for 40 min. Cells were then fixed with 2% PFA for 10 min at room temperature. Primary antibodies used in this study were mouse anti-GFP (Roche; 11814460001, batch 11063100, dilution 1:1,000), rabbit anti-paxillin (Abcam; ab32084, batch GR23669-20, dilution 1:250), mouse anti-VSV-G (a gift from T. Kreis, University of Geneva; dilution 1:500), mouse anti-GM130 (BD Biosciences; 610823, batch 4324839, dilution 1:1,000), human anti-RAB6:GTP (AA2; Adipogen; dilution 1:250), mouse monoclonal anti-SBP tag (clone 20; Millipore; dilution 1:400), rabbit polyclonal anti-GFP (Recombinant Antibody Platform, Institut Curie; dilution 1:400), monoclonal mouse anti-GFP (Roche; 11814460001, dilution 1:1,000), anti–α-tubulin (Sigma-Aldrich; t6199, dilution 1:1,000), polyclonal rabbit anti-RAB6 (Santa Cruz; sc-310, dilution 1:1,000), and rabbit anti-ELKS (Monier et al., 2002, 1:1,000 or Proteintech; 22211-1-AP, 1:5,000). Alexa Fluor- and HRP–coupled secondary antibodies were purchased from Jackson ImmunoResearch Laboratory. Coverslips were mounted in Mowiol and examined under a 3D deconvolution microscope (Leica DM-RXA2), equipped with a piezo z-drive (Physik Instrument) and a 100× 1.4NA-PL-APO objective lens for optical sectioning. 3D or 1D multicolor image stacks were acquired using Metamorph software (MDS) through a cooled charge-coupled device (CCD) camera (Photometrics Coolsnap HQ). Pictures were acquired in a quantitative manner using the same exposure settings for the different conditions of a same experiment.

### Live-cell imaging

Spinning-disk confocal time-lapse imaging was done at 37°C in a thermostat-controlled chamber using an Eclipse 80i microscope (Nikon) equipped with spinning disk confocal head (Perkin), a 100× objective, and either an Ultra897 iXon camera (Andor) or CoolSnapHQ2 camera (Roper Scientific). Fixed samples were imaged using a 60× objective with the same setup. TIRF microscopy was done using Leibovitz’s medium (Life Technologies) at 37°C in a thermostat-controlled chamber. An Eclipse Ti inverted microscope (Nikon) equipped with either a TIRF module (Nikon) or an iLAS2 azimuthal TIRF module (Roper Scientific), a 100× TIRF objective, a beam splitter (Roper Scientific), and an Evolve 512 electron-multiplying CCD camera (Photometrics) was used in this case (Boulanger et al., 2014). All acquisitions were driven by Metamorph (Molecular Devices).

### FACS

Cells were incubated at 4°C with a rabbit anti-GFP antibody diluted in PBS (Recombinant Protein Platform, Institut Curie, 1:400). Intra- and extracellular EGFP intensity was detected with a BD Accuri C6 Cytometer. The ratio between the cell surface signal intensity per the total signal intensity detected in SBP-EGFP-PLAP expressing cells measured at 30 and 60 min was normalized to the cell surface signal intensity detected at 0 min (corresponding to background) for each condition.

### SUnSET assay

SUnSET was performed as described previously (Schmidt et al., 2009). Briefly, MEF cells were seeded at a confluence of 30% and then treated with ethanol (control) or 1 µM of 4-OH for 96 h to deplete RAB6. The day of the experiment, cells were cultured without serum and incubated for 30 min at 37°C with 10 µg/ml of puromycin. Puromycin was then chased at different time points (0, 1, 2, 4, and 5.5 h). The supernatants and the whole-cell lysates were collected and processed for Western blotting. The puromycin signal was revealed using a mouse anti-puromycin antibody (clone 12D10; Millipore; MABE343). HRP-conjugated anti-mouse secondary antibody–associated signal was detected with the enhanced chemiluminescence system (ChemiDoc Touch System; Bio-Rad). Quantification of puromycin intensity in the supernatant and the whole-cell lysis from four independent experiments using Image Lab software (Bio-Rad) are shown. The amount of total secreted proteins was determined by the normalization of the intensity of puromycin signal in the supernatant for each time point by the sum of the intensity of puromycin signal in the supernatant and the whole-cell lysate for each corresponding time point.

### Coimmunoprecipitation and Western blotting

To test the interaction between GFP-RAB6A, GFP-RAB6A-S52D, and endogenous ELKS, HeLa cells transfected with GFP, GFP-RAB6A or GFP-RAB6A-S52D were trypsinized, washed once in PBS, and incubated on ice for 60 min in a lysis buffer: 25 mM Tris, pH 7.5, 100 mM NaCl, and 0.1% NP-40. Cells were centrifuged for 10 min at 10,000 *g* to remove cell debris, and extracts were processed for coimmunoprecipitation using GFP-trap (Chromotek) following the manufacturer’s instructions.

For Western blotting experiments, cells were washed three times in ice-cold PBS, scraped, and then lysed in a buffer containing 150 mM NaCl, 50 mM Tris-HCl, pH 7.5, and 1% NP-40 (Sigma-Aldrich). Protein concentrations were determined by Quick Start Bradford 1× Dye Reagent (Bio-Rad). Equal amounts of proteins were reduced with 1× loading buffer containing 6% β-mercaptoethanol and resolved on 10% SDS-PAGE. Proteins were transferred onto nitrocellulose Protran BA 83 membranes (Life Science) and processed for immunoblotting. HRP-conjugated secondary antibody–associated signal was detected with the ECL system (ChemiDoc Touch System, Bio-Rad). Quantification of the corresponding signal obtained was done with Image laboratory software.

ColX secretion following RAB6 depletion was measured using Western blotting. After release of the cargo from the ER for different time points, trafficking was stopped by incubating the cells at 4°C, and the supernatant was collected. Secreted ColX was concentrated using a Vivaspin Turbo 4 column (filters <10 kD). When the culture media volume reached 65 µl, the centrifugation was stopped, and 5× loading buffer containing 6% β-mercaptoethanol was added. Cells were lysed in 150 mM NaCl, 50 mM Tris-HCl, pH 7.5, and 1% Nonidet-P40, and proteins were boiled at 95°C for 5 min. Samples were then processed for Western blotting.

The following primary antibodies were used: mouse anti-GFP (Roche; 11814460001, batch 11063100, dilution 1:1,000), rabbit anti-ELKS (Monier et al., 2002, 1:1,000 or Proteintech; 22211-1-AP, 1:5,000), polyclonal rabbit anti-RAB6 (Santa Cruz; sc-310, dilution 1:1,000), monoclonal anti-actin (Sigma-Aldrich, clone AC-40, catalog number A4700, dilution 1:5,000), and monoclonal anti-tubulin (Recombinant Antibody Platform, Institut Curie; 1:500).

### Image analysis and quantification

For Figs. 1 A, 2 (D–F), and S5 D, temporal projections were performed with the tool Temporal-Color Code in Fiji software. For Fig. 2 H, intensity profiles were calculated from a drawing line with Fiji software.

The quantification of proximity between secreted cargos and FAs was performed using MatLab. The quantification method was developed by Quantacell (France), a company specialized in bioimage analysis and statistics. Objects were segmented using thresholding and Gaussian filtering, and the region of interest corresponding to the whole cell was drawn manually. The distance of each pixel of a segmented object corresponding to secreted cargo (green channel) to the closest pixel from FAs (red channel) was measured. As a baseline for comparison of different samples, the distance of any pixel of the region of interest corresponding to the closest pixel of FAs was measured. The ratio of enrichment compared with the distance of pixels of the region of interest to the closest FAs was calculated and is displayed in Fig. 2 D. Kymographs of Fig. 2 (F and H) were done on Fiji software. Quantification of colocalization in post-Golgi carriers (Fig. 4, E and G; Fig. 6, A and B; and Figs. S3 C, S4, and S5, C and D) was performed manually. Colocalization between two moving vesicles (or fixed vesicles in Fig. 6 B) in two different channels was determined manually using Synchronize Windows in ImageJ software (National Institutes of Health). In Fig. S4 B, colocalization was measured with Pearson’s coefficient using ImageJ. Quantification of the velocity and the track length (the covered distance) of vesicles was done with the plug-in Manual Tracking in ImageJ, developed by Fabrice Cordelières (Institut Curie). The number of vesicles was determined using ImageJ using the Find Maxima plug-in. The number of vesicles was normalized either per cell or per area in each cell.

For quantification of the number of FAs (Fig. S2) and number and intensity of secreted spots (Fig. 3 G) of specific cargos, the threshold was adjusted for each image using ImageJ or Fiji. The number of FAs and the number of secreted spots were calculated following manual selection using ImageJ or Fiji. To calculate the intensity at secreted spots, the mean fluorescence intensity of secreted spots was calculated from data collected from threshold images. Then normalization was performed by dividing, for each cell, the mean intensity at secretion spots by the total mean intensity of the cell in the same channel.

### Statistical analysis

All data were generated from cells pooled from at least three independent experiments represented as n. In Fig. 3 G, a representative experiment from three independent experiments is presented. Statistical data are presented as means ± SEM. Statistical significance was determined by Student’s *t* test for two or three sets of data using Excel; no sample was excluded. Cells were randomly selected. P < 0.05 was considered statistically significant.

### Online supplemental material

Fig. S1 shows validation of the SPI assay, visualization of the whole ventral plasma membrane by TIRF, and arrival at the plasma membrane after the induction of secretion without SPI. Fig. S2 shows synchronized transport of SBP-EGFP-gp135 in cells coated on different substrates and the effect of control, ELKS, KIF5B, RAB6, and RAB8A siRNAs on the size of FAs. Fig. S3 characterizes the RAB6A-S52D mutant. Fig. S4 shows colocalization of RAB6 with the GPI-APs between ER–Golgi and Golgi to plasma membrane and of the cargos with endogenous RAB6:GTP. Fig. S5 shows that RAB6 depletion affects PLAP arrival at the plasma membrane; myosin II–dependent fission of EGFP-CD59–positive vesicles from Golgi membranes; SUnSET assay for quantification of protein secretion in MEF RAB6^lox/lox^ cells; and effect of mCherry-RAB6wt and mCherry-RAB6-S52D on protein secretion. Fig. S6 shows that RAB6 and RAB8 are colocalized on post-Golgi carriers. RAB6 and RAB8 act in the same post-Golgi trafficking pathway. Video 1 shows the synchronized secretion of SBP-EGFP-ColX. Video 2 shows the synchronized secretion of SBP-EGFP-ColX using the SPI assay.

## Supplementary Material

Supplemental Materials (PDF)

Video 1

Video 2
