# Supplementary material for: RAB6 and microtubules restrict protein secretion to focal adhesions
Source: J Cell Biol. 2019 May 29;218(7):2215–31. doi: 10.1083/jcb.201805002 (PMC6605799; doi:10.1083/jcb.201805002)
Supplement: Supplemental Materials (PDF) [file JCB_201805002_sm.pdf]

Supplemental material

Fourriere et al., https://doi.org/10.1083/jcb.201805002

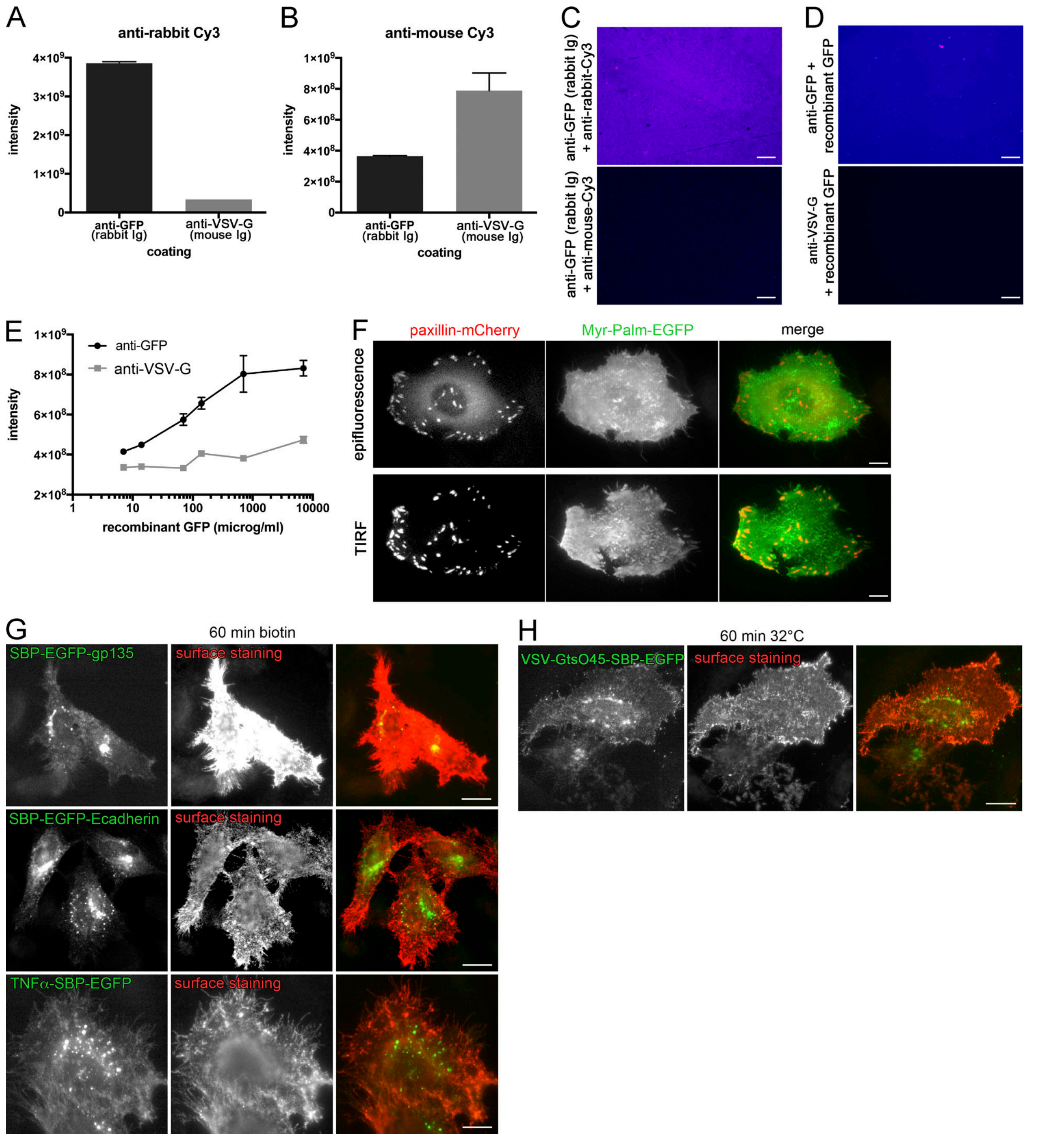

Figure S1. **Validation of the SPI assay and visualization of the whole ventral plasma membrane by TIRF. Arrival at the plasma membrane after the induction of secretion without SPI. (A–C)** Glass coverslips were coated with either anti-GFP antibodies (rabbit Ig) or anti–VSV-G antibodies (mouse Ig). Secondary anti-rabbit or anti-mouse antibodies conjugated to Cy3 were used to detect the primary antibodies coated. **(C)** Fields of view of coverslips coated with anti-GFP and revealed as indicated. The same parameters of acquisition were used for the two fields of view. Fire lookup table was used (generated with Fiji). Error bars represent mean ± SEM. **(D and E)** Glass coverslips were coated with either anti-GFP antibodies or anti–VSV-G antibodies as control. Serial dilutions of recombinant GFP were incubated onto these coated coverslips. GFP fluorescence signal was quantitated (arbitrary units). **(E)** Fields of view of coverslips coated with anti-GFP (top) or anti–VSV-G (bottom) antibodies and incubated with recombinant GFP at 700 µg/ml. Fire lookup table was used (generated with Fiji). **(F)** The same cell overexpressing paxillin-mCherry to visualize FAs and myristoylated and palmitoylated EGFP to visualize the plasma membrane was imaged using a microscope in either epifluorescence or TIRF mode. **(G)** Induction of secretion using the RUSH system. HeLa cells stably expressing Str-KDEL were transfected with TNFα-SBP-EGFP, SBP-EGFP-Ecadherin, or SBP-EGFP-gp135. Induction of trafficking by addition of biotin was performed at time 0. Surface staining was performed using an anti-GFP antibody (red) on nonpermeabilized cells. **(H)** Trafficking induction of the thermo-sensitive mutant VSV-G. HeLa cells were transfected with VSV-G tsO45-EGFP. Induction of trafficking by a temperature switch from 40°C to 32°C was performed at time 0. Surface staining was performed using an anti–VSV-G antibody (red) on nonpermeabilized cells.
Scale bars, 10 µm.

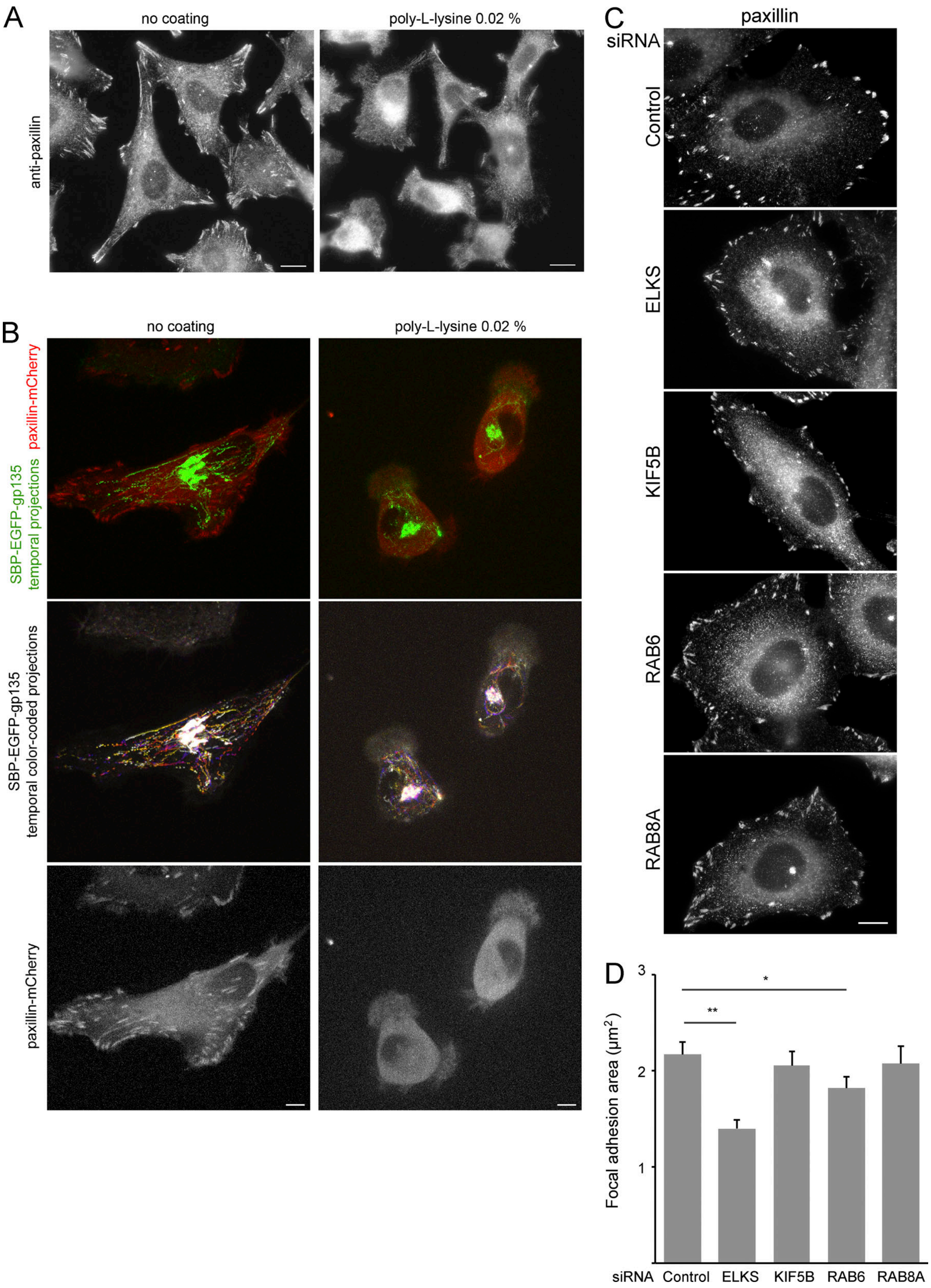

Figure S2. **Synchronized transport of SBP-EGFP-gp135 in cells coated on different substrates; effect of control, ELKS, KIF5B, RAB6, and RAB8A siRNAs on the size of FAs. (A)** HeLa cells seeded on glass coverslips either noncoated or coated with poly-L-lysine (0.02%). Focal adhesions were immunolabeled using an anti-paxillin antibody. **(B)** HeLa cells expressing paxillin-mCherry and Str-KDEL-SBP-EGFP-gp135 were seeded on glass coverslips either noncoated or coated with poly-L-lysine (0.02%). The transport of post-Golgi carriers containing SBP-EGFP-gp135 was monitored ~20 min after biotin addition. Temporal projections were obtained using the Fiji plugin Temporal-Color Code. **(C)** HeLa cells treated with control, ELKS, KIF5B, RAB6, or RAB8A siRNAs for 72 h were fixed and stained for paxillin. Representative images of cells treated with the different siRNAs and stained with an anti-paxillin antibody. **(D)** Quantification of the FA area (in μm²) of cells treated as described in A (mean ± SEM, *n* = 3). *, $P < 0.05$; **, $P < 10^{-4}$ (Student's *t* test). Scale bars, 10 μm.

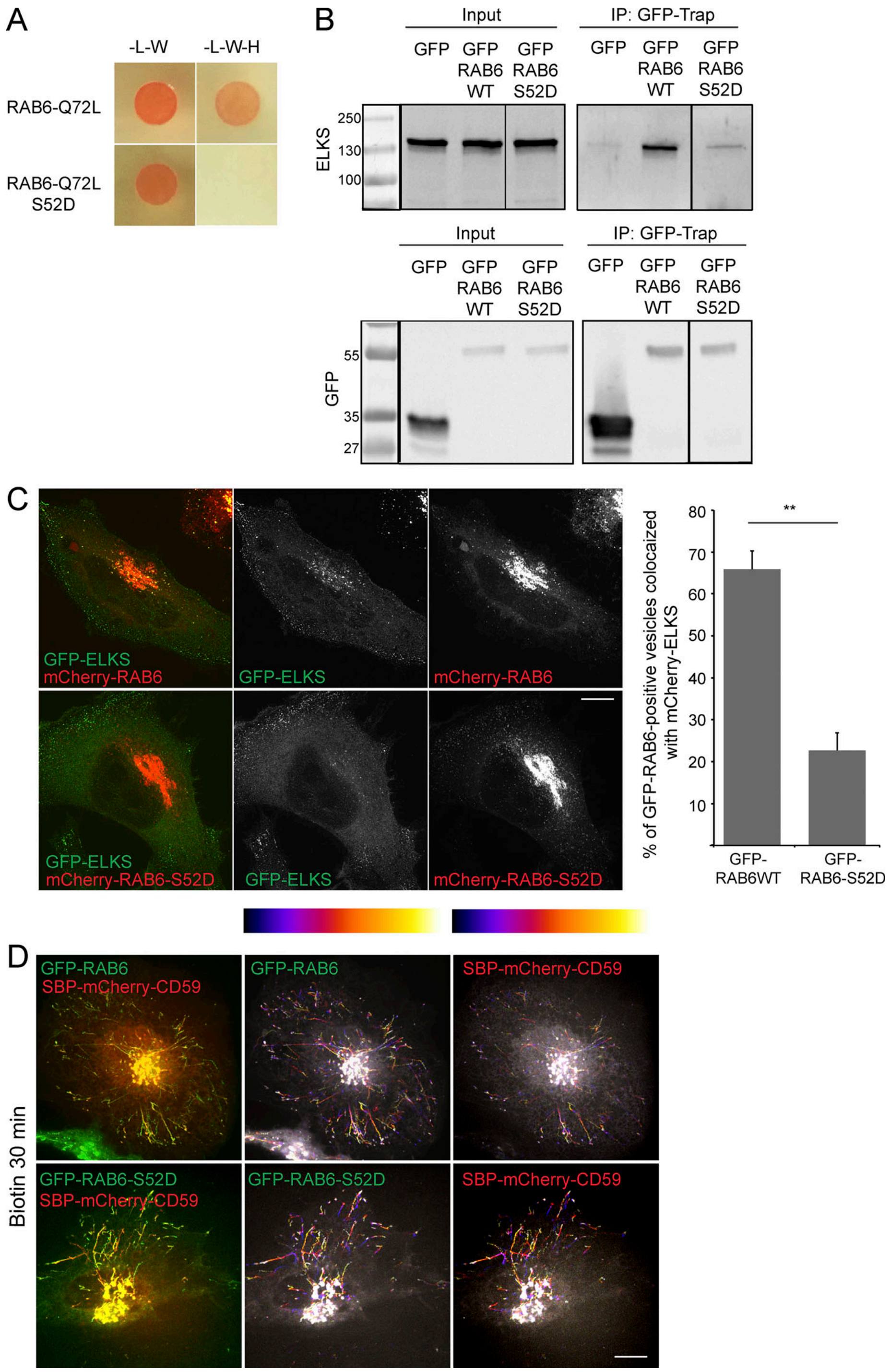

Figure S3. **Characterization of the RAB6A-S52D mutant. (A)** Yeast two-hybrid interactions between the RAB6-binding domain of ELKS (Monier et al., 2002) and RAB6A-Q72L WT or S52D. The *S. cerevisiae* reporter strain L40 was cotransformed with a plasmid encoding fusion proteins to detect interactions between RAB6-binding domain of ELKS and RAB6A-Q72L or RAB6A-Q72L-S52D. Growth on medium lacking histidine (-W-L-H) indicates an interaction between the encoded proteins. **(B)** Endogenous ELKS is pulled down by GFP-RAB6A and not by GFP-RAB6A-S52D. GFP-RAB6A, GFP-RAB6A-S52D, or GFP-transfected HeLa cell extracts were immunoprecipitated using the GFP-trap system. ELKS bound to GFP-RAB6 was revealed by Western blot analysis using anti-ELKS antibody. GFP, GFP-RAB6A, and GFP-RAB6A-S52D were revealed by anti-GFP antibody. Input represents a 5% load of the total cell extracts used in all conditions. **(C)** Left: HeLa cells coexpressing GFP-ELKS with mCherry-RAB6 or mCherry-RAB6-S52D were fixed and imaged. Right: Quantification of the number of GFP-RAB6– or GFP-RAB6-S52D–positive vesicles colocalizing with mCherry-ELKS (mean ± SEM, $n = 3$). **, $P < 0.005$ (Student's t test). **(D)** HeLa cells were co-transfected with mCherry-CD59 and GFP-RAB6 or GFP-RAB6-S52D. Cells were observed by time-lapse imaging using a spinning disk microscope. Biotin was added at time 0. After a 30-min incubation with biotin, mCherry-CD59 localizes in the Golgi apparatus, and fast acquisition imaging was performed. A temporal projection of mCherry-CD59 and GFP-RAB6A WTor S52D signal over a 60-s video was performed using Fiji software and is represented with a temporal color code (top of each image). GFP-RAB6 and GFP-RAB6-S52D were colocalized on post-Golgi tracks exiting the Golgi complex en route to the plasma membrane. Scale bars, 10 µm.

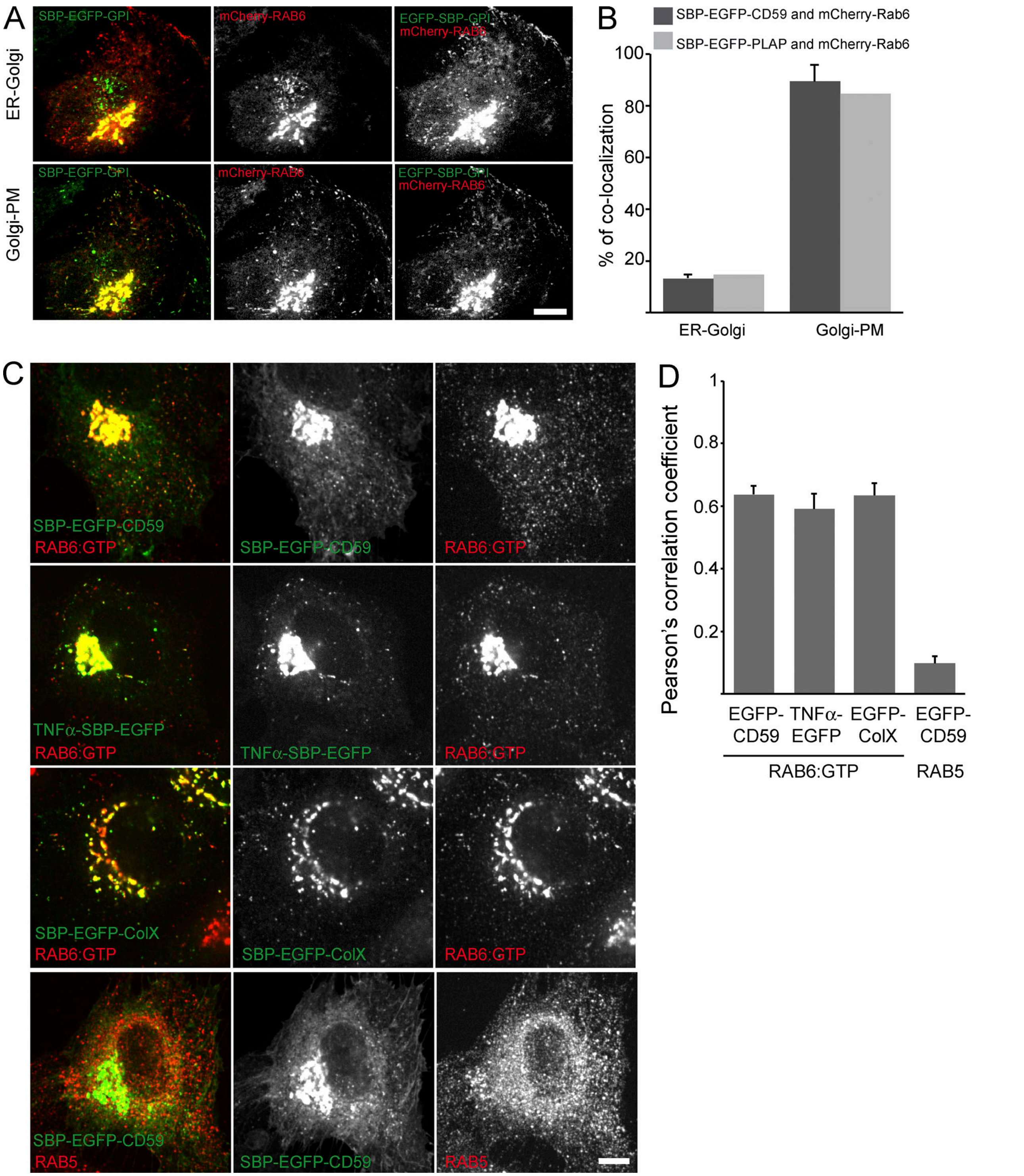

Figure S4. **Colocalization of RAB6 with the GPI-APs between ER-Golgi and Golgi to plasma membrane; colocalization of the cargos with endogenous RAB6:GTP. (A)** RPE-1 cells coexpressing mCherry-RAB6 with SBP-EGFP-CD59 or SBP-EGFP-PLAP were incubated for 10 min (to monitor ER to Golgi transport) or 30 min (to monitor Golgi to plasma membrane transport) with biotin and imaged using time-lapse video-microscopy. Representative images taken from videos of cells expressing SBP-EGFP-GPI are displayed. **(B)** Quantification of the percentage of colocalization between mCherry-RAB6 and SBP-EGFP-CD59 or SBP-EGFP-PLAP at 10 and 30 min after biotin addition (mean ± SEM, *n* = 2–12 cells). Scale bar: 10 µm. **(C)** Staining for endogenous RAB6:GTP of HeLa or RPE-1 cells expressing SBP-EGFP-CD59, TNFα-SBP-EGFP, or SBP-EGFP-ColX incubated with biotin for 30 min. Staining for endogenous RAB5 of HeLa cells expressing SBP-EGFP-CD59 and incubated with biotin for 30 min. Scale bar: 10 µm. **(D)** Colocalization (Pearson's coefficient) between RAB6, RAB5, and the cargos (mean ± SEM, *n* = 35–39 regions from 12 cells).

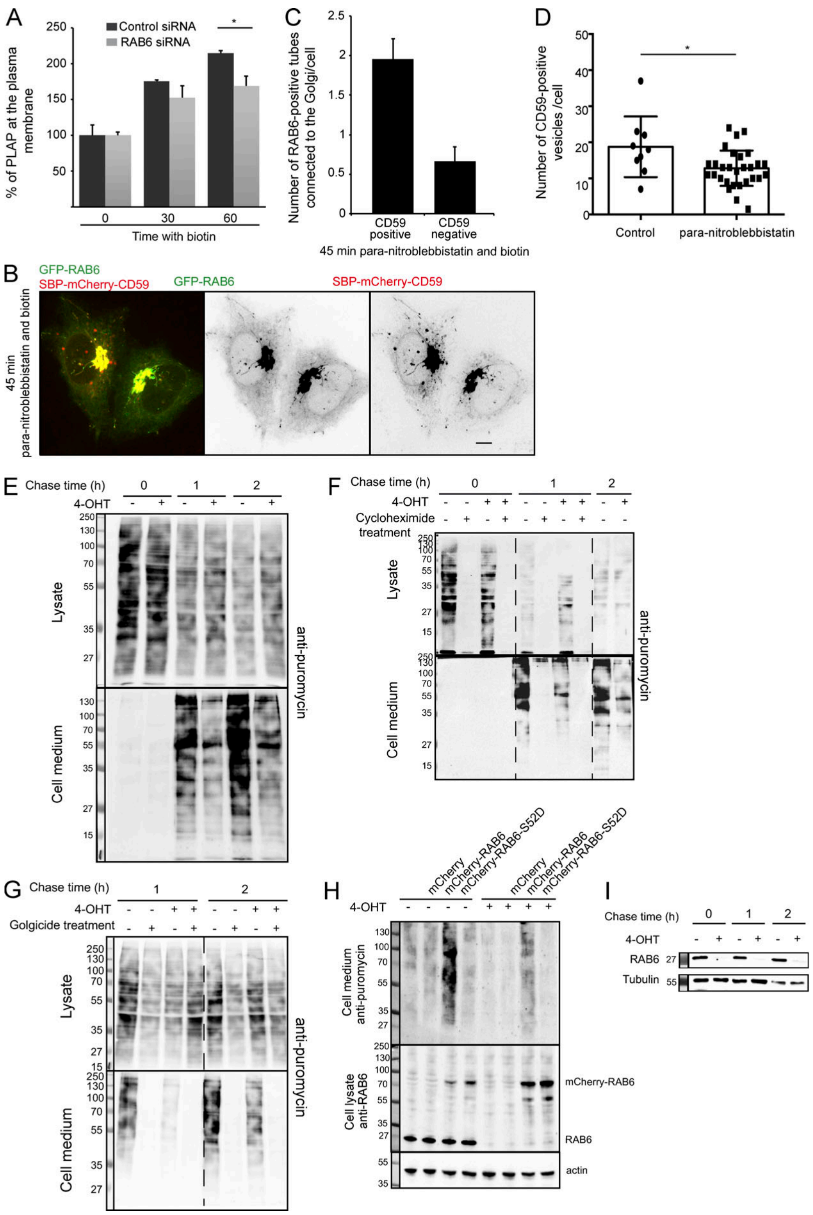

Figure S5. **RAB6 depletion affects PLAP arrival at the plasma membrane; myosin II–dependent fission of EGFP-CD59–positive vesicles from Golgi membranes; SUnSET assay for quantification of protein secretion in MEF RAB6$^{lox/lox}$ cells; and effect of mCherry-RAB6wt and mCherry-RAB6-S52D on protein secretion. (A)** Quantification of SBP-EGFP-PLAP at the cell surface by FACS in cells transfected with control or RAB6 siRNA and incubated for 30 or 60 min with biotin (mean ± SEM, n = 3). P < 0.05 (Student's t test). After a 60-min incubation with biotin, we observed a 50% decrease in the arrival of PLAP at the plasma membrane. *, P < 0.05 (Student's t test). **(B)** HeLa cells coexpressing GFP-RAB6 and SBP-mCherry-CD59 were treated for 45 min with para-nitroblebbistatin. After 15 min, biotin was added in the medium to allow cargo release from the ER, and cells were imaged using time-lapse video-microscopy. Representative images taken from videos. Higher magnification on the right. Scale bar, 10 µm. **(C)** Quantification of the number of tubes connected to the Golgi complex containing RAB6 with or without CD59 in cells treated as indicated in B (mean ± SEM, n = 10 cells). **(D)** Quantification of the number of transport carriers in cells treated as indicated in B (mean ± SEM, n = 9–29 cells). *, P < 0.05 (Student's t test). **(E)** MEFs prepared from RAB6 loxP/KO Rosa26CreERT2-TG embryos (described in Bardin et al. [2015]; named after MEF RAB6$^{lox/lox}$) were treated with ethanol or 4-OHT for 96 h to induce RAB6 depletion. Cells were then incubated with puromycin and chased in puromycin-free medium for 0, 1, 2, 4, and 5.5 h. Total proteins in cell lysates and supernatants (medium) were labeled with an anti-puromycin antibody. Representative immunoblots are displayed. **(F)** MEF RAB6$^{lox/lox}$ were treated with ethanol or with 4-OHT for 96 h to induce RAB6 depletion. Cells were treated with cycloheximide for 15 min, then incubated for 30 min with puromycin and chased in a medium without puromycin for 0, 1, and 2 h. Total proteins in cell lysates and supernatants were labeled with an anti-puromycin antibody. Representative immunoblots are displayed. **(G)** MEFs RAB6$^{lox/lox}$ were treated with ethanol or 4-OHT for 96 h. Cells were treated with Golgicide A for 30 min, then incubated for 30 min with puromycin and chased in a medium without puromycin for 0, 1, and 2 h. Total proteins in cell lysates and supernatants were labeled with an anti-puromycin antibody. Representative immunoblots are displayed. **(H)** MEF RAB6$^{lox/lox}$ cells were transfected with mCherry, mCherry-RAB6, or mCherry-RAB6-S52D and treated with ethanol or 4-OHT for 96 h. Cells were then incubated with puromycin and chased in puromycin-free medium for 1 h. Total proteins in cell supernatants (medium) were labeled with anti-puromycin. Cell lysates were incubated with anti-RAB6 antibody. Actin was used as a loading control. Representative immunoblots are displayed. **(I)** MEF cells treated as indicated in A were subjected to Western blot analysis. RAB6 was revealed using anti-RAB6 antibody. Tubulin signal was used as a loading control.

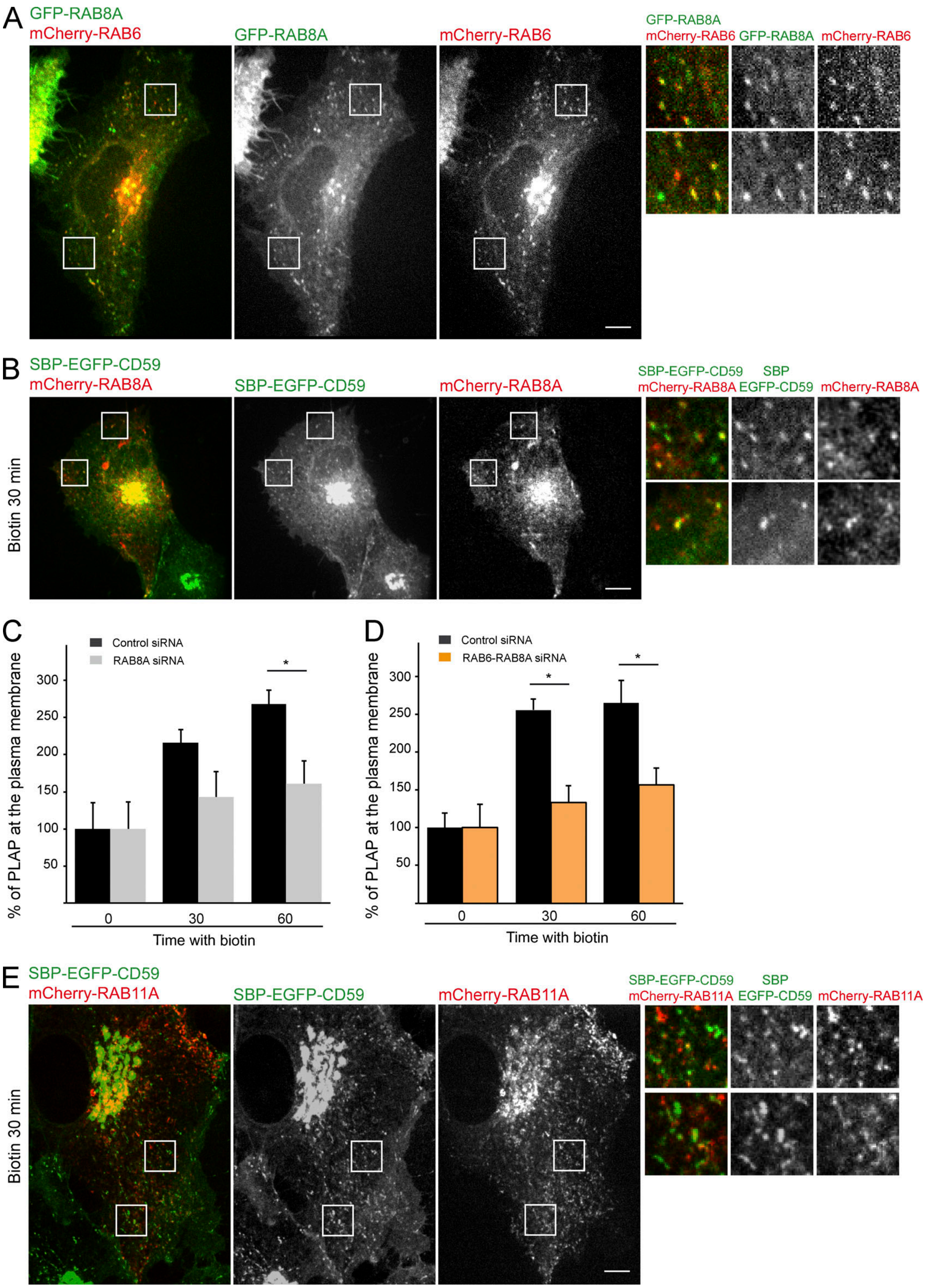

Figure S6. **RAB6 and RAB8 are found colocalized on post-Golgi carriers; RAB6 and RAB8 act in the same post-Golgi trafficking pathway. (A)** HeLa cells coexpressing GFP-RAB8A and mCherry-RAB6 were imaged using a time-lapse spinning-disk confocal microscope. Representative images taken from videos are displayed. **(B)** RPE1-SBP-EGFP-CD59 coexpressing mCherry-RAB8A were incubated for 30 min with biotin to allow cargo release from the ER. Cells were imaged using a time-lapse spinning-disk confocal microscope. Representative images taken from videos are displayed. **(C)** Quantification of SBP-EGFP-PLAP at the cell surface by FACS in cells transfected with control or RAB8A siRNA and incubated for 30 or 60 min with biotin (mean ± SEM, $n = 3$). *, $P < 0.05$ (Student's *t* test). After a 60-min incubation with biotin, we observed a 50% decrease in the arrival of PLAP at the plasma membrane. **(D)** Quantification of SBP-EGFP-PLAP at the cell surface by FACS in cells transfected with control or RAB6 and RAB8A siRNA and incubated for 30 or 60 min with biotin (mean ± SEM, $n = 3$). *, $P < 0.05$ (Student's *t* test). After a 30- or 60-min incubation with biotin, we observed a 50% decrease in the arrival of PLAP at the plasma membrane. **(E)** RPE1-SBP-EGFP-CD59 coexpressing mCherry-RAB11A were incubated for 30 min with biotin to allow cargo release from the ER. Cells were imaged using a time-lapse spinning-disk confocal microscope. Representative images taken from videos are displayed. Scale bars, 10 µm.

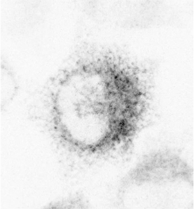

Video 1. **Synchronized secretion of SBP-EGFP-ColX.** HeLa cells stably expressing SBP-EGFP-ColX were observed in real time using a spinning disk microscope. A projection of several Z-slices is shown. Acquisition was performed every 40 s for 36 min, and biotin was added at frame 2. Corresponds to Fig. 1 A.

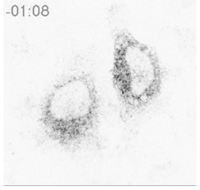

Video 2. **Synchronized secretion of SBP-EGFP-ColX using the SPI assay.** HeLa cells stably expressing SBP-EGFP-ColX and seeded on anti-GFP–coated coverslips (SPI assay) were observed in real time using a spinning disk microscope. A projection of several Z-slices is shown. Time is indicated in minutes:seconds. Biotin was added at frame 2 (00:00). Corresponds to Fig. 1 C.
